# Multivariate assessment of event-related potentials with the t-CWT method

**DOI:** 10.1186/s12868-015-0185-z

**Published:** 2015-11-05

**Authors:** Vladimir Bostanov

**Affiliations:** Institute of Medical Psychology and Behavioral Neurobiology, University of Tübingen, Gartenstr. 29, 72074 Tübingen, Germany

**Keywords:** Event-related brain potentials, ERP, Continuous wavelet transform, CWT, t-CWT, Principal component analysis, PCA, Multivariate statistics

## Abstract

**Background:**

Event-related brain potentials (ERPs) are usually assessed with univariate statistical tests although they are essentially multivariate objects. Brain–computer interface applications are a notable exception to this practice, because they are based on multivariate classification of single-trial ERPs. Multivariate ERP assessment can be facilitated by feature extraction methods. One such method is t-CWT, a mathematical-statistical algorithm based on the continuous wavelet transform (CWT) and Student’s t-test.

**Results:**

This article begins with a geometric primer on some basic concepts of multivariate statistics as applied to ERP assessment in general and to the t-CWT method in particular. Further, it presents for the first time a detailed, step-by-step, formal mathematical description of the t-CWT algorithm. A new multivariate outlier rejection procedure based on principal component analysis in the frequency domain is presented as an important pre-processing step. The MATLAB and GNU Octave implementation of t-CWT is also made publicly available for the first time as free and open source code. The method is demonstrated on some example ERP data obtained in a passive oddball paradigm. Finally, some conceptually novel applications of the multivariate approach in general and of the t-CWT method in particular are suggested and discussed.

**Conclusions:**

Hopefully, the publication of both the t-CWT source code and its underlying mathematical algorithm along with a didactic geometric introduction to some basic concepts of multivariate statistics would make t-CWT more accessible to both users and developers in the field of neuroscience research.

**Electronic supplementary material:**

The online version of this article (doi:10.1186/s12868-015-0185-z) contains supplementary material, which is available to authorized users.

## Background

An event-related brain potential (ERP) is extracted from an electroencephalogram (EEG) [1]. Since the EEG is a stochastic process, the ERP is a multivariate statistical object, as well. It is a set of random curves (one curve per EEG channel), and a random curve cannot be simply represented by a single univariate feature (or a curve parameter) without loosing a lot of potentially useful information. Nevertheless, ERPs are usually represented by univariate “components”, which most often are prominent peaks of the curves. An ERP component is usually assessed by its peak value or by the area under the curve in a narrow time window around the peak. This method has the huge advantage that it is both simple and visually and intuitively clear. For instance, peak latency can be interpreted as brain processing time related to an ERP-triggering event. More sophisticated methods for ERP component extraction from single trials exist as well [2]. The statistical assessment of ERP components is usually based on analysis of variance (ANOVA) [3]. Such assessment is sufficient for many applications. Some inherent limitations of this approach have been addressed by “mass univariate analysis” methods based on permutation/randomization tests [4, 5].

There are, however, cases in which univariate assessment is not sufficient. For instance, ERP-based brain–computer interfaces (BCI) require full-fledged multivariate assessment of the ERP curves [6]. The t-CWT [7], a feature extraction method based on the continuous wavelet transform (CWT) [8, 9] and Student’s t-test, was first introduced as one possible multivariate solution to the problem of single-trial ERP classification at the International BCI Competition 2003, where it was compared to a variety of other feature extraction and classification methods ranging from simple and weak to advanced and powerful ones like, e.g., Support Vector Machines (SVM) [6, 10]. t-CWT was a winner on two of the six ERP datasets provided for the competition [6]; these two were obtained in two different BCI paradigms, “P300 Speller” and “Self regulation of Slow Cortical Potentials (Biofeedback)” [7].

Apart from BCI applications, it has been shown how t-CWT can be used for detection and quantification of ERPs in other paradigms as well, e.g., for individual (clinical) diagnostics [11, 12]. In the latter application, t-CWT was combined with a multivariate randomization test based on Hotelling’s T${}^2$-statistic. Similarly to the univariate randomization tests mentioned above [4], which provide an effective correction for multiple univariate comparisons, the multivariate randomization test [11] corrects the accumulation of chance bias arising from performing both t-CWT feature extraction and hypothesis testing on the same ERP dataset.

A comparison of t-CWT to classical univariate ERP assessment methods based on peak picking and area computation showed a clear advantage of multivariate t-CWT features over univariate measures [11]. More recently, t-CWT has been systematically evaluated in comparison to several other ERP component detection methods ranging from simple univariate peak picking to $t_\mathrm {max}$ randomization tests [4] performed on band pass filtered EEG and on t-CWT features [13]. This evaluation was performed with both simulated and real ERP data at different levels of signal-to-noise ratio. Sensitivity, specificity, positive and negative predictive values were assessed, and, as a result, t-CWT showed superior to all other methods, especially at low signal-to-noise ratios. It should be noted, however, that, in that study, t-CWT was used only as a feature extraction method and only mass univariate analysis ($t_\mathrm {max}$), but no multivariate statistical assessment of the obtained features was done. In another recent study, t-CWT feature extraction was compared to two other wavelet-based ERP component detection methods using the spikelet technique and wavelet asymmetry, respectively, with the result that both t-CWT and the novel wavelet asymmetry method showed a marked advantage over the spikelet technique in terms of detection accuracy: 83 % and 91 % vs. 43 %, correspondingly [14]. In the latter study, however, only a single ERP component of interest, the N160, was detected without any multivariate or mass univariate analyses of the whole ERP curves.

Although t-CWT features may well be interpreted as ERP components [7, 11, 14], the current article is mainly focused on t-CWT applications in which, as in the case of BCI, the whole (multivariate) difference between two ERP curves is of primary importance, while differences in single components are only of secondary interest.

The goals of the this article are: (a) to provide a didactic geometric primer on some basic concepts of multivariate statistics as applied to ERP assessment in general and to the t-CWT method in particular; (b) to present for the first time a detailed, step-by-step, formal description of the t-CWT algorithm and to make its MATLAB and GNU Octave [15] implementation publicly available as free and open source code [16, 17] (Additional file 1) released under the GNU General Public License, version 3 (GPLv3, full text available in the t-CWT documentation [17]); (c) to demonstrate the t-CWT method in the assessment of example ERP data [18] obtained in a passive oddball paradigm [19, 20]; (d) to suggest and discuss conceptually novel applications of the multivariate approach in general and of the t-CWT method in particular for the purpose of hypothesis testing rather than for BCI and single-trial classification.

An important new pre-processing step in the revised version of the t-CWT algorithm presented here is the novel multivariate outlier rejection procedure based on principal component analysis (PCA, see below). Other changes in the algorithm were aimed at simplification for the sake of easier understanding. For instance, the time-dependent filtering module [7] was removed from the current version and the discrete wavelet transform (DWT) [8, 21] was replaced by a discrete Fourier transform (DFT) [22]. Visualization was simplified as well by plotting the one-dimensional linear discriminant function (LDF, see below) instead of the two-dimensional t-value scalogram [7].

### Multivariate statistics: a geometric primer

Multivariate statistical analysis [23] cannot be substituted by a multitude of univariate analyses, because the latter cannot address adequately the covariance of the data. Even if the effect of the correlations between the variables on multiple univariate tests is taken into account by a proper correction of the overall $\alpha$-level, e.g., by a permutation test [4], the univariate approach still misses a lot of information contained in the covariance matrix [23, p. 8].

In this section, some basic concepts of multivariate analysis are represented as geometric notions. This approach is meant as an aid to better understanding of these concepts through there association with familiar images from Euclidean space.

#### ERPs as points in a vector space

Consider a sample of *N* ERP trials obtained from *K* EEG channels, in the time interval (epoch) $0\le t<T$, where *t* is the time relative to a triggering event. For each channel $k=1,2,\ldots K$, and each single trial $n=1,2,\ldots N$, the ERP voltage curve $v_n^k(t)$ can be represented by a row vector $\mathbf {v}^k_n=\left( v_n^{k1},v_n^{k2},\ldots v_n^{kL}\right)$ with components $v_n^{kl} = v_n^k(t^l)$, where $t^l=(l-1)T/L$, $l=1,2,\ldots L$ are the equidistant sampling time points whose number *L* is obtained from the original sampling frequency $R_0$:

1 $$\begin{aligned} L=R_0 T. \end{aligned}$$

The entire *K*-channel ERP trial can be represented by the vector

2 $$\begin{aligned} \mathbf {v}_n = \left( \mathbf {v}^1_n,\mathbf {v}^2_n,\ldots \mathbf {v}^K_n\right) . \end{aligned}$$

Thus, the ERP is represented by a random vector $\mathbf {v}$ in a $K\times L$-dimensional space, and the ERP sample is represented by the *N*-by-$K\times L$ matrix

3 $$\begin{aligned} \mathbf {V} = \begin{pmatrix} \mathbf {v}_1 \\ \mathbf {v}_2 \\ \vdots \\ \mathbf {v\!}_N \end{pmatrix}. \end{aligned}$$

Note that this notation is different from the standard notation in which vectors are represented by columns [23, p. 7]. Here, vectors are represented by rows, as in the MATLAB and GNU Octave [15] implementation of t-CWT [16, 17].

#### Mahalanobis distance and Hotelling’s test

Now, consider a random vector $\mathbf {v}$ and the corresponding single-trial ERP sample $\mathbf {V}$ which, however, in this case, comprises two subsamples $\mathbf {V}_{\!\!_\mathcal {A}}$ and $\mathbf {V}_{\!_\mathcal {B}}$ obtained under two different experimental conditions $\mathcal {A}$ and $\mathcal {B}$ ($\mathcal {A}$-$\mathcal {B}$ design). The two corresponding ERPs are then represented by $\mathbf {V}_{\!\!_\mathcal {A}}$ and $\mathbf {V}_{\!_\mathcal {B}}$ and by the respective random vectors $\mathbf {v}_{\!\!_\mathcal {A}}$ and $\mathbf {v}_{\!_\mathcal {B}}$. Assuming multivariate normal distributions [23, pp. 37–59] with equal covariance matrices, we want to compare the two ERPs by testing the hypothesis $H_0: \mathbf {\overline{v}}_{\!\!_\mathcal {A}}= \mathbf {\overline{v}}_{\!_\mathcal {B}}$, where $\mathbf {\overline{v}}_{\!\!_\mathcal {A}}$ and $\mathbf {\overline{v}}_{\!_\mathcal {B}}$ are the mean vectors (representing the average ERP curves). Geometrically, $\mathbf {\overline{v}}_{\!\!_\mathcal {A}}$ and $\mathbf {\overline{v}}_{\!_\mathcal {B}}$ represent two points in the $K\times L$-dimensional space. Hence, we can test $H_0$ by testing the hypothesis that the *distance* between these two points is zero. Thus, the problem of multivariate hypothesis testing can be reduced to the geometric problem of defining and measuring distance in this vector space and then performing a univariate test on this (random) distance.

Like in the univariate case, the scale for measuring distance is provided by the variance or, more precisely, by its square root, the standard deviation (like in Student’s t-test). There are, however, two problems in the multivariate case: (a) we have different variances corresponding to the different vector components, and (b) these variables are correlated. The latter problem can be solved by principal component analysis (PCA). The principal component transform (PCT) represented by its corresponding matrix $\mathbf {T_p}$ is an orthogonal transformation, i.e., a kind of rotation of the coordinate axes, such that, in the new coordinate system, the covariance matrix has a diagonal form [23, pp. 343–344]. The transformation of the vector components is given by

4 $$\begin{aligned} \mathbf {v_p} = \mathbf {v}\mathbf {T_p},\quad \mathbf {V\!_p} =\mathbf {V}\mathbf {T_p}. \end{aligned}$$

The covariance matrix $\mathbf {S}$ is diagonalized by this transformation, i.e., the correlations between the new variables $v^i_\mathrm {p},\ i=1,2,\ldots K\times L$ are all zero. Because $\mathbf {T_p}$ is an orthogonal transformation, the inverse transformation (i.e., the rotation back to the original axes) is given by the transpose

5 $$\begin{aligned} \mathbf {v} = \mathbf {v_p}\,\mathbf {T_p^{-1}} = \mathbf {v_p}\,\mathbf {T_p^\mathsf {T}},\quad \mathbf {V} = \mathbf {V\!_p}\,\mathbf {T_p^\mathsf {T}}, \end{aligned}$$

and, correspondingly, the diagonalized covariance matrix $\mathbf {S_p}$ is given by

6 $$\begin{aligned} \mathbf {S_p} = \mathbf {T_p^\mathsf {T}}\,\mathbf {S}\,\mathbf {T_p},\quad \mathbf {S} = \mathbf {T_p}\,\mathbf {S_p}\,\mathbf {T_p^\mathsf {T}}. \end{aligned}$$

The diagonal elements (the eigenvalues) of $\mathbf {S_p}$ are the squared standard deviations $\left( \sigma ^i_\mathrm {p}\right) ^2$ of the uncorrelated variables $v^i_\mathrm {p}$. Normalizing these variables by the transformation

7 $$\begin{aligned} x^i=v^i_\mathrm {p}/\sigma ^i_\mathrm {p}, \end{aligned}$$

we land in a familiar $K\times L$-dimensional Euclidean space where the scale is the same in all directions, i.e., the constant density ellipsoids [23, p. 40] of the multivariate normal distribution of $\mathbf {x}$ are spheres (the procedure (4–7) is known as PCA “sphering” or “whitening”). In Euclidean space, the squared distance $D^2$ between two points $\mathbf {x\,}_{\!\!_\mathcal {A}}$ and $\mathbf {x\,}_{\!_\mathcal {B}}$ is simply given by the Pythagorean theorem

8 $$\begin{aligned} D^2(\mathbf {x\,}_{\!\!_\mathcal {A}},\mathbf {x\,}_{\!_\mathcal {B}}) = \sum _{i=1}^{K\times L} \left( x^i_{\!\!_\mathcal {A}}- x^i_{\!_\mathcal {B}}\right) ^2. \end{aligned}$$

Substituting (7) into (8), we can compute the distance between $\mathbf {\overline{v}_{p\,{\!\!_\mathcal {A}}}}$ and $\mathbf {\overline{v}_{p\,{\!_\mathcal {B}}}}$

9 $$\begin{aligned} D^2(\mathbf {\overline{v}_{p\,{\!\!_\mathcal {A}}}},\mathbf {\overline{v}_{p\,{\!_\mathcal {B}}}}) = \left( \mathbf {\overline{v}_{p\,{\!\!_\mathcal {A}}}} - \mathbf {\overline{v}_{p\,{\!_\mathcal {B}}}} \right) \, \mathbf {S_p^{-1}}\, \left( \mathbf {\overline{v}_{p\,{\!\!_\mathcal {A}}}} - \mathbf {\overline{v}_{p\,{\!_\mathcal {B}}}} \right) ^\mathsf {T}. \end{aligned}$$

In (9), we use the fact that $\mathbf {S_p^{-1}}$ is a diagonal matrix with eigenvalues $\left( \sigma ^i_\mathrm {p}\right) ^{-2}$. Substituting the right parts of (5) and (6) into (9), we obtain

10 $$\begin{aligned} D^2( \mathbf {\overline{v}}_{\!\!_\mathcal {A}}, \mathbf {\overline{v}}_{\!_\mathcal {B}}) = \left( \mathbf {\overline{v}}_{\!\!_\mathcal {A}}- \mathbf {\overline{v}}_{\!_\mathcal {B}}\right) \, \mathbf {S^{-1}}\, \left( \mathbf {\overline{v}}_{\!\!_\mathcal {A}}- \mathbf {\overline{v}}_{\!_\mathcal {B}}\right) ^\mathsf {T}. \end{aligned}$$

In (10), we use the property that $\mathbf {S^{-1}}$ is diagonalized by the same transformation as $\mathbf {S}$. The expression (10) is called a “Mahalanobis distance” [23, p. 22]. When $\mathbf {S}$ is substituted with an unbiased estimator and the resulting $D^2$ is multiplied by a proper coefficient (a function of the degrees of freedom), it turns into Hotelling’s T${}^2$-statistic whose *p*-value is obtained from the corresponding (univariate) T${}^2$-distribution and can be used to test $H'_0: D^2( \mathbf {\overline{v}}_{\!\!_\mathcal {A}}, \mathbf {\overline{v}}_{\!_\mathcal {B}}) = 0$, which is equivalent to $H_0: \mathbf {\overline{v}}_{\!\!_\mathcal {A}}= \mathbf {\overline{v}}_{\!_\mathcal {B}}$ [23, pp. 60–120]. Thus, the multivariate problem of comparing two mean vectors (representing two average ERP curves) is reduced to the univariate problem of testing whether the Mahalanobis distance between the mean vectors is different from zero.

#### Linear discriminant analysis (LDA)

So far, we have shown that Hotelling’s T${}^2$ is a kind of Mahalanobis distance between two mean vectors defined by the natural metric provided by the covariance. But (10) bears another geometric interpretation as well. Defining the linear discriminant function (LDF) $\mathbf {d}$ [23, pp. 74] as a *column* vector by

11 $$\begin{aligned} \mathbf {d} = \mathbf {S^{-1}}\, \left( \mathbf {\overline{v}}_{\!\!_\mathcal {A}}- \mathbf {\overline{v}}_{\!_\mathcal {B}}\right) ^\mathsf {T}, \end{aligned}$$

the Mahalanobis distance (10) is expressed as a LDF value:

12 $$\begin{aligned} D^2( \mathbf {\overline{v}}_{\!\!_\mathcal {A}}, \mathbf {\overline{v}}_{\!_\mathcal {B}}) = \left( \mathbf {\overline{v}}_{\!\!_\mathcal {A}}- \mathbf {\overline{v}}_{\!_\mathcal {B}}\right) \,\mathbf {d}. \end{aligned}$$

The column vector $\mathbf {d}$ defines a direction in space. The projection of the mean difference $\mathbf {\overline{v}}_{\!\!_\mathcal {A}}- \mathbf {\overline{v}}_{\!_\mathcal {B}}$ onto this particular direction is the maximum of all projections of $\mathbf {\overline{v}}_{\!\!_\mathcal {A}}- \mathbf {\overline{v}}_{\!_\mathcal {B}}$ onto all possible directions [23, pp. 92]. Linear discriminant analysis (LDA) is based on the construction of a separation plane which is perpendicular to $\mathbf {d}$ and passes through the middle between $\mathbf {\overline{v}}_{\!\!_\mathcal {A}}$ and $\mathbf {\overline{v}}_{\!_\mathcal {B}}$, i.e. through the point $(\mathbf {\overline{v}}_{\!\!_\mathcal {A}}+\mathbf {\overline{v}}_{\!_\mathcal {B}})/2$. This separation plane is defined by the equation

13 $$\begin{aligned} \mathbf {v}\mathbf {d} = \tfrac{1}{2}(\mathbf {\overline{v}}_{\!\!_\mathcal {A}}+\mathbf {\overline{v}}_{\!_\mathcal {B}})\,\mathbf {d}. \end{aligned}$$

According to the optimal LDA classification rule [23, pp. 231], an ERP trial $\mathbf {v}_{\!x}$ drawn from a new sample $\mathbf {V\!}_\mathcal {X}$ (comprising an unknown mixture of vectors drawn from both populations $\mathcal {A}$ and $\mathcal {B}$) is assigned to the population $\mathcal {A}$ if it lies above this plane, i.e.

14 $$\begin{aligned} \mathbf {v}_{\!x} \mathbf {d} > \tfrac{1}{2}(\mathbf {\overline{v}}_{\!\!_\mathcal {A}}+\mathbf {\overline{v}}_{\!_\mathcal {B}})\,\mathbf {d}, \end{aligned}$$

and it is assigned to the population $\mathcal {B}$ otherwise. The rule (14) is optimal because it minimizes the classification error rate.

Moreover, the LDF value difference $\left( \mathbf {v}_{\!x}\!- \tfrac{1}{2}(\mathbf {\overline{v}}_{\!\!_\mathcal {A}}\!+\mathbf {\overline{v}}_{\!_\mathcal {B}}) \right) \mathbf {d}$ is proportional to the distance from the point $\mathbf {v}_{\!x}$ to the separation plane (13) and can be seen as a measure of the affiliation of $\mathbf {v}_{\!x}$ with $\mathcal {A}$ or $\mathcal {B}$.

The optimal LDA classification rule (14) is based on the assumption that the a priori probability $p_{\!\!_\mathcal {A}}$ that an ERP trial of unknown affiliation belongs to $\mathcal {A}$ is equal to the *a priori* probability $p_{\!_\mathcal {B}}$ that it belongs to $\mathcal {B}$. If $p_{\!\!_\mathcal {A}}\!\ne p_{\!_\mathcal {B}}$, the optimal LDA classification rule (14) is generalized as follows [23, pp. 231]. An ERP trial $\mathbf {v}_{\!x}$ is assigned to $\mathcal {A}$ if

15 $$\begin{aligned} \mathbf {v}_{\!x}\mathbf {d} > \tfrac{1}{2}(\mathbf {\overline{v}}_{\!\!_\mathcal {A}}+\mathbf {\overline{v}}_{\!_\mathcal {B}})\,\mathbf {d} +\ln \left( \frac{p_{\!_\mathcal {B}}}{p_{\!\!_\mathcal {A}}}\right) , \end{aligned}$$

and to $\mathcal {B}$ otherwise, i.e., the separation plane (13) is shifted in the direction of $\mathbf {\overline{v}}_{\!\!_\mathcal {A}}$ if $p_{\!\!_\mathcal {A}}\!< p_{\!_\mathcal {B}}$, and it is shifted in the direction of $\mathbf {\overline{v}}_{\!_\mathcal {B}}$ if $p_{\!\!_\mathcal {A}}\!> p_{\!_\mathcal {B}}$. Note that (15) is a generalization of (14) because the logarithmic term is zero when $p_{\!\!_\mathcal {A}}\!=p_{\!_\mathcal {B}}$.

The LDF value $\mathbf {v}_{\!x}\mathbf {d}$ has an interesting property that should be noted, because it could be important for some interesting applications. Since the LDF $\mathbf {d}$ is obtained from the sample $\mathbf {V}$ while $\mathbf {v}_{\!x}$ is drawn from a different sample $\mathbf {V\!}_\mathcal {X}$, the LDF value $\mathbf {v}_{\!x}\mathbf {d}$ has univariate normal distribution [23, p. 45]. This property can be used to reduce the multivariate ERP $\mathbf {v}_{\!x}$ to the univariate random variable $\mathbf {v}_{\!x}\mathbf {d}$ that exclusively and fully reflects the (multivariate) ERP difference between two particular experimental conditions, $\mathcal {A}$ and $\mathcal {B}$. The important point here is that $\mathbf {v}_{\!x}\mathbf {d}$ can be used for other purposes beside classification. For instance, if the $\mathcal {A}$-$\mathcal {B}$ structure of $\mathbf {V\!}_\mathcal {X}$ is known, the LDF value $\mathbf {v}_{\!x}\mathbf {d}$ can be subjected to Student’s t-test in order to test the hypothesis about the mean $\mathcal {A}$-$\mathcal {B}$ difference within $\mathbf {V\!}_\mathcal {X}$. Note the difference to Hotelling’s $T^2$-test: while in the Mahalanobis distance (12), the LDF $\mathbf {d}$ is computed from the sample that is tested, in $\mathbf {v}_{\!x}\mathbf {d}$ it is computed from a different sample. This means that the multivariate *structure* of the $\mathcal {A}$-$\mathcal {B}$ difference derived from $\mathbf {V\!}$ is imposed on $\mathbf {V\!}_\mathcal {X}$ in order to assess the (univariate) *magnitude* of the $\mathcal {A}$-$\mathcal {B}$ difference within $\mathbf {V\!}_\mathcal {X}$. This procedure provides a methodologically important alternative to Hotelling’s $T^2$-test, because it implies a conceptually novel approach to multivariate ERP assessment which could be useful, e.g., in clinical applications (see the “Discussion” section).

**Fig. 1:**
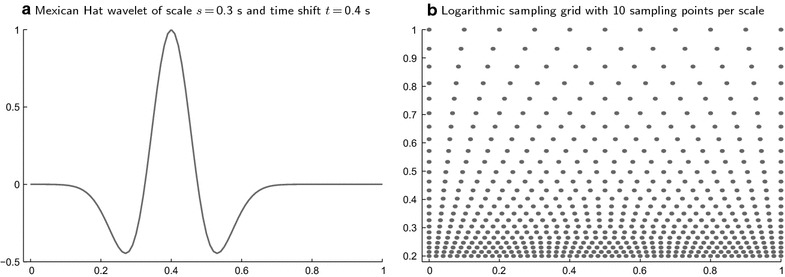
A Mexican Hat wavelet and a logarithmic sampling grid. The *left plot* **a** displays a Mexican Hat wavelet as a function of time (in seconds). The scale (approximate wavelength) is $s=0.3$ s and the time shift (the position of the maximum) is $t=0.4$ s. The *right plot* **b** displays a log-grid in the *t*-*s*-plane (*t* at the horizontal axis, *s* at the vertical axis, both measured in seconds). The scale-invariant sampling rate is $R=10$ pps. Note that the number of sampling points in the time interval $0 \le t < 0.2$ at $s=0.2$ is the same, as in the interval $0 \le t < 0.3$ at $s=0.3$, and as in the interval $0 \le t < 0.4$ at $s =0.4$, etc., and as the number of lines of points in the scale interval $0.2 \le s < 0.4$, which in turn is the same as in the interval $0.4 \le s < 0.8$, etc.

#### The problem of dimensionality

The multivariate ERP model presented above is idealized. It can only work with few EEG channels, low time-sampling rates, short epochs and large number of ERP trials. In most applications, however, the dimensionality of the vector space is very large and exceeds the number of trials. For instance, if the number of channels is *K* = 32, the sampling rate (1) is $R_0$ = 500 Hz, and the epoch length is *T* = 1 s, then the number of time points is *L* = $R_0 T$ =500, and we have $K\times L$ = 16,000 dimensions. If the number of trials is, e.g., *N* = 1,000, the rank of the covariance matrix $\mathbf {S}$ is $N-1=999$ [23, pp. 9, 406], which means that $\mathbf {S}$ is singular (i.e. $\mathbf {S^{-1}}$ does not exist, because $\mathbf {S}$ has at least 15,001 zero eigenvalues), and (9–15) cannot be applied. Even if $N-1>K\times L$ and $\mathbf {S}$ is not singular, it is still necessary to reduce the dimensionality of the model because of (a) the loss of statistical power caused by the inclusion of noise variables in the $D^2$-sum (9), and (b) the computational problems associated with too many variables and too small eigenvalues.

A standard solution to the dimensionality problem is given by PCA (see above): the variables $v^i_\mathrm {p}$ with small variances $\left( \sigma ^i_\mathrm {p}\right) ^2$ are simply deleted from the model according to a certain criterion, e.g., all eigenvalues greater than the average are retained, or the largest eigenvalues explaining a certain proportion of the total variance are retained [23, pp. 347–348].

The t-CWT method provides a further solution to the dimensionality problem. It uses explicitly the special fact that the random vectors represent ERP curves, i.e., *continuous* functions of time. The continuous wavelet transform (CWT) [8, 9] and Student’s t-test are used to extract certain “features” of the ERP curves, which build the “feature space” whose dimensionality is substantially smaller than that of the original space. All the standard multivariate procedures described above (PCA, Hottelling’s test, LDA) can then be performed in this feature space.

Here, it is interesting to mention an alternative, recently proposed, dimensionality reduction approach based on Effect-Matched Spatial (EMS) filtering [24]. While t-CWT reduces the dimensionality of the ERP time curves, independently for each single channel, the EMS filtering method reduces the number of channels (to one), independently for each single time point, thus representing the multichannel ERP by a single “surrogate time-course”. Thus, in a certain sense, EMS filtering can be seen as complementary to t-CWT and, for particular purposes, the two methods can be used in combination with each other.

### The t-CWT method

The t-CWT method has already been described in the continuous notation [7]. Here, it is presented in the discrete vector and matrix notation as well, because this discrete representation is the one that is used in the computational algorithm [16, 17].

#### CWT

The CWT [8, 9] $w^k_n(s,t)$ of the EEG signal $v^k_n(t)$ of the *k*th channel of the *n*th ERP trial is given by

16 $$\begin{aligned} w^k_n(s,t) = \frac{1}{\sqrt{s}}\int \limits _0^T v^k_n(\tau ) \psi \left( \frac{\tau -t}{s}\right) d\tau , \end{aligned}$$

where $\psi (t)$ is a wavelet function which is (a) well localized in both time and frequency, and (b) has a zero mean:

17 $$\begin{aligned} \int \limits _{-\infty }^\infty \psi (t)dt=0. \end{aligned}$$

The approximate position of $\psi \big ((\tau -t)/s\big )$ in time is determined by the time shift *t*, while the scale *s*, which is the inverse frequency, defines the approximate position in the frequency domain (Fig. 1a).

The CWT (16) is a linear transformation, which means that it can be represented by a matrix $\mathbf {T_w}$ such that

18 $$\begin{aligned} \mathbf {w} = \mathbf {v}\mathbf {T_w},\quad \mathbf {W} =\mathbf {V}\mathbf {T_w}, \end{aligned}$$

where the random vectors $\mathbf {v}$ and $\mathbf {w}$ represent the ERP in the time domain and in the time-frequency domain, respectively, and the matrices $\mathbf {V}$ and $\mathbf {W}$ represent the corresponding single-trial samples. The coefficients of $\mathbf {T_w}$ in (18) are obtained by substituting $v^k_n(t)$ and $\psi \big ((\tau -t)/s\big )$ in (16) with their respective discrete representations as in (2) and converting the integrals into corresponding sums. Note, however, that the CWT (16) is highly redundant and the wavelet space defined by $\mathbf {w}$ is much “larger” than the original vector space defined by $\mathbf {v}$. Note also that $\mathbf {T_w}$ is actually a block diagonal matrix built from *K* identical blocks, one per channel. The t-CWT computer application [16, 17] uses only one CWT block which is applied to each channel.

#### t-CWT

Now, consider again two experimental conditions $\mathcal {A}$ and $\mathcal {B}$ ($\mathcal {A}$-$\mathcal {B}$ design), and the corresponding two samples of sizes *M* and *N*, respectively, of *K*-channel ERPs represented by the random curves $v^k_{{\!\!_\mathcal {A}}m}(t)$ and $v^k_{{\!_\mathcal {B}}n}(t)$, where: $k=1,2,\ldots K$; $m=1,2,\ldots M$; $n=1,2,\ldots N$; and $0\le t<T$. The corresponding CWTs are computed by (16). Then, Student’s two-sample t-value $\mathtt {t}^k(s,t)$ is computed for each *k* and each scale-time point (*s*, *t*) from the corresponding CWT values $w^k_{{\!\!_\mathcal {A}}m}(s,t)$ and $w^k_{{\!_\mathcal {B}}n}(s,t)$:

19 $$\begin{aligned} \mathtt {t}^k(s,t) = \sqrt{\frac{MN}{M+N}}\ \frac{\overline{w}^k_{\!\!_\mathcal {A}}(s,t) - \overline{w}^k_{\!_\mathcal {B}}(s,t)}{\sigma ^k_{{\!\!_\mathcal {A}}{\!_\mathcal {B}}}(s,t)}, \end{aligned}$$

where $\overline{w}^k_{\!\!_\mathcal {A}}(s,t)$ and $\overline{w}^k_{\!_\mathcal {B}}(s,t)$ are the sample means and $\sigma ^k_{{\!\!_\mathcal {A}}{\!_\mathcal {B}}}(s,t)$ is the pooled standard deviation computed from the corresponding sums of squares (SS).

In the next step, each of the points $(s^{kj},t^{kj})$, at which the functions $\mathtt {t}^k(s,t)$ reach a local extremum, are detected. In the last step, we define the t-CWT vector samples $\mathbf {W}^\star _{\!\!_\mathcal {A}}$ and $\mathbf {W}^\star _{\!_\mathcal {B}}$ by their respective components, the t-CWT features $w_{{\!\!_\mathcal {A}}m}^{\star kj}$ and $w_{{\!_\mathcal {B}}n}^{\star kj}$ defined by

20 $$\begin{aligned} w_{{\!\!_\mathcal {A}}m}^{\star kj} = w^k_{{\!\!_\mathcal {A}}m}(s^{kj},t^{kj}),\quad w_{{\!_\mathcal {B}}n}^{\star kj} = w^k_{{\!_\mathcal {B}}n}(s^{kj},t^{kj}). \end{aligned}$$

Finding the local extrema of a function of two variables is an analytical operation, but its result can be represented by a simple projection in the wavelet space, i.e. selecting the vector components that correspond to the points $(s^{kj},t^{kj})$ and discarding all other dimensions. This projection can be represented by the matrix $\mathbf {T_w^\star }$ which is obtained from $\mathbf {T_w}$ in (18) by deleting the columns corresponding to the discarded space dimensions. The t-CWT vectors are obtained by substituting $\mathbf {T_w}$ in (18) with $\mathbf {T_w^\star }$

21 $$\begin{aligned} \mathbf {w^\star } = \mathbf {v}\mathbf {T_w^\star },\quad \mathbf {W^\star } =\mathbf {V}\mathbf {T_w^\star }, \end{aligned}$$

where $\mathbf {V}$ is the “total” ERP sample comprising the subsamples $\mathbf {V}_{\!\!_\mathcal {A}}$ and $\mathbf {V}_{\!_\mathcal {B}}$, and $\mathbf {v}$ is the corresponding random vector.

## Methods

This section provides a detailed, step-by-step, formal delineation of the t-CWT algorithm. In the brief intuitive descriptions published before [7, 11], most of the details were omitted. Here, a rigorous mathematical delineation of all steps is presented for the first time.

### Pre-processing

Theoretically, the t-CWT could be performed directly in the time domain defined by (2). The CWT (16–18) is, however, highly redundant and computationally demanding. That is why the dimensionality of the vector space must be reduced substantially before computing $\mathbf {w}$ and $\mathbf {w^\star }$ (16–21).

#### Frequency domain representation

The first pre-processing step is based on a frequency domain representation of the ERPs by a discrete Fourier transform (DFT [22]; note the difference to the previous version of t-CWT [7] in which time-dependent filtering and DWT [8, 21] were performed instead of DFT). The dimensionality of the vector space is reduced by deleting all frequencies larger than $2f_c$, where $f_c$ is a cutoff frequency defined by a cutoff scale

22 $$\begin{aligned} S_c=1{/}f_c. \end{aligned}$$

This is done as follows. First, we compute the orthogonal (real) DFT matrix $\mathbf {T_f}$. The ERP vectors (2–3) are then transformed by

23 $$\begin{aligned} \mathbf {v_f} = \mathbf {v}\mathbf {T_f},\quad \mathbf {V\!_f} =\mathbf {V}\mathbf {T_f}. \end{aligned}$$

Geometrically, the DFT (23) can be seen as a rotation of the axes, analogical to the PCT (4). Note, however, that $\mathbf {T_f}$ is actually a block diagonal matrix built from zeros and *K* identical DFT blocks, one per channel. (The t-CWT computer application uses only one copy of the DFT block which is applied to each channel.)

As next, we retain only those columns of $\mathbf {T_f}$ that correspond to frequencies $f^j$ fulfilling the cutoff condition

24 $$\begin{aligned} f^j\le 2f_c=\frac{2}{S_c},\quad j=1,2,\ldots \end{aligned}$$

A “reduced” matrix ${\hat{{\mathbf {T}}}}_{\mathbf {f}}$ is obtained from $\mathbf {T_f}$ by deleting all columns corresponding to frequencies $f^j>2f_c$. The reduced DFT is then given by

25 $$\begin{aligned} \hat{\mathbf {{v}}}_{\mathbf {f}} = {\mathbf {v}}\hat{\mathbf {{T}}}_{\mathbf {f}},\quad \hat{\mathbf {{V}}}_{\mathbf {f}} =\mathbf {V}\hat{\mathbf {{T}}}_{\mathbf {f}}. \end{aligned}$$

As in (5), the “inverse” transform (i.e., the rotation back to the time domain axes) is given by

26 $$\begin{aligned} \hat{\mathbf {{v}}} =\hat{\mathbf {{v}}}_{\mathbf {f}}\,\hat{\mathbf {{T}}}_{\mathbf {f}}^ {\mathsf {T}},\quad \hat{\mathbf {{V}}} = \hat{\mathbf {{V}}}\!_{\mathbf {f}}\,\hat{\mathbf {{T}}}_{\mathbf {f}}^{\mathsf {T}}. \end{aligned}$$

Note, however, that, since $\hat{\mathbf {{T}}}_{\mathbf {f}}$ is not a square matrix, $\hat{\mathbf {{v}}}$ and $\hat{\mathbf {{V}}}$ are filtered versions of $\mathbf {v}$ and $\mathbf {V}$.

In order to smooth the cutoff, the vector components corresponding to the frequencies $f^j$ of the last octave $f_c<f^j\le 2f_c$ are attenuated gradually. This is done by multiplication with a diagonal matrix $\mathbf {R_f}$ whose diagonal elements are given by the values of an envelope function *r*(*f*) such that $r(f^j)=1$, for $f^j\le f_c$, and $r(f^j)=2-f^j\!/\!f_c$, for $f_c<f^j\le 2f_c$. Similarly, the vector components in the time domain, $\mathbf {v}$ and $\mathbf {V}$, can also be multiplied with an appropriate window function before DFT. This is done by left multiplication of $\hat{\mathbf {{T}}}_{\mathbf {f}}$ with a diagonal matrix $\mathbf {R_t}$ whose diagonal elements are given by the values of the corresponding envelope function. The current t-CWT implementation uses a modified Tukey window [25] defined by the envelope function

27 $$\begin{aligned} f(t) = \left\{ \begin{array}{l@{\quad }l} \frac{1}{2}\Big (1-\cos \left( \pi \frac{T_\mathrm {in}}{T}\right) \Big ) \!&{}\! \text {for } 0 \le t < T_\mathrm {in} \\ 1 \!&{}\! \text {for } T_\mathrm {in} \le t < T_\mathrm {out} \\ \frac{1}{2}\Big (1-\cos \left( \pi \frac{T - T_\mathrm {out}}{T}\right) \Big ) \!&{}\! \text {for } T_\mathrm {out} \le t < T \end{array}\right. \end{aligned}$$

where $T_\mathrm {in}$ is the fade-in time and $T_\mathrm {out}$ is the fade-out time.

Chaining all transformations together, we obtain

28 $$\begin{aligned} \tilde{\mathbf {{v}}}_{\mathbf {f}} = \mathbf {v}\tilde{\mathbf {{T}}}_{\mathbf {f}},\quad \tilde{\mathbf {{V}}}_{\mathbf {f}} =\mathbf {V}\tilde{\mathbf {{T}}}_{\mathbf {f}}, \end{aligned}$$

where $\tilde{\mathbf {{T}}}_{\mathbf {f}}$ is defined by

29 $$\begin{aligned} \tilde{\mathbf {{T}}}_{\mathbf {f}} = \mathbf {R_t}\,\hat{\mathbf {{T}}}_{\mathbf {f}}\,\mathbf {R_f}. \end{aligned}$$

For some purposes (e.g. visualization), it might be useful to represent the filtered ERP back in the time domain by the “inverse” transform (26):

30 $$\begin{aligned} \tilde{\mathbf {{v}}} = \tilde{\mathbf {{v}}}_{\mathbf {f}}\hat{\mathbf {{T}}}_{\mathbf {f}}^{\mathsf {T}} = \mathbf {v}\tilde{\mathbf {{T}}}_{\mathbf {f}}\hat{\mathbf {{T}}}_{\mathbf {f}}^{\mathsf {T}}. \end{aligned}$$

The number of frequency components per channel is

31 $$\begin{aligned} N\!_F = 1 + 4f_c T, \end{aligned}$$

where *T* is the length of the time interval. The dimension of the frequency domain space (i.e., the number of rows of $\hat{\mathbf {{T}}}_{\mathbf {f}}$ or $\tilde{\mathbf {{T}}}_{\mathbf {f}}$) is then $KN\!_F$, where K is the number of EEG channels. Substituting (22) in (31) we obtain the following approximation:

32 $$\begin{aligned} N\!_F\approx 4\,\frac{T}{S_c} \end{aligned}$$

In the t-CWT software [16, 17], the time-to-frequency domain transformation (28) is implemented by the function tcwt_t2f; the preceding computation of the transformation matrix $\tilde{\mathbf {{T}}}_{\mathbf {f}}$ (22–29) is implemented by the function tcwt_prm2mat (Table 1).

**Table 1:** A schematic representation of the t-CWT algorithm as implemented by the software [16, 17]

**t-CWT**	**Input**	**Processing**	**Output**
**Function**	**Files**	**Steps**	**Files: variables (equations)**
prm2mat	*input parameter file*	Computes time-to-frequency domain transformation matrix, CWT matrix and log-grid matrix	_A_const.mat : $\tilde{\mathbf {T}}_{\mathbf {f}}$ (22–29), $\tilde{\mathbf {T}}_{\mathbf {w}}$ (16–18, 43–45), $(s^g,t^{g,h})$ (40–41)
t2f	* .t.mat	Performs time-to-frequency domain transformation and filtering of ERP datasets	* .f.mat : $\tilde{\mathbf {V}}\!_{\mathbf {f}}$ (28)
	_A_const.mat		* .ri0.mat : *initial index vector*
f2pc	* .f.mat	Computes PCT by performing PCA-based multivariate outlier detection in frequency domain	* .pc.mat : ${\hat{\mathbf {T}}}_{\mathbf {p}}$ (33–38)
	* .ri0.mat		* .ri1.mat : *outlier index vector*
f2cwss	* .f.mat	Performs CWT and computes sums of squares (SS) for Student’s t-values for t-CWT scalograms	* .cwss.mat : SS of ${\tilde{\mathbf {W}}}$ (48) for the computation of $\sigma ^k_{{\!\!_\mathcal {A}}{\!_\mathcal {B}}}(s,t)$ and $\mathtt {t}^k(s,t)$ (19)
	* .pc.mat		
	* .ri1.mat		
	_A_const.mat		
pc2cnd2ri	* .f.mat	Performs outlier detection for each experimental condition separately with fixed PCT obtained by f2pc	* .ri2.mat : *outlier index vector*
	* .pc.mat		
	* .ri1.mat		
f2x	* .f.mat	Computes t-CWT scalograms using SS computed by f2cwss, detects t-CWT scalogram extrema, and computes t-CWT matrix	* .tcw.mat : $\mathtt {t}^k(s,t)$ (19)
	* .pc.mat		* .x.mat : $\tilde{\mathbf {T}}^\star _{\mathbf {w}}$ (49)
	* .cwss.mat		
	* .ri2.mat		
	_A_const.mat		
x2ld	* .f.mat	Performs PCA-based step-down reduction of t-CWT features obtained by f2x and computes LDF in reduced feature space	* .ld.mat : ${\hat{\mathbf {T}}}^{\mathbf {\star }}_{\mathbf {p}}$ (50–51), $\mathbf {d_f^\star }$ (52–55)
	* .x.mat		
	* .ri2.mat		

The full names of the functions include the prefix ‘tcwt_’ (e.g., ‘tcwt_t2f’). The functions listed above (except for prm2mat) operate according to the following general scheme:

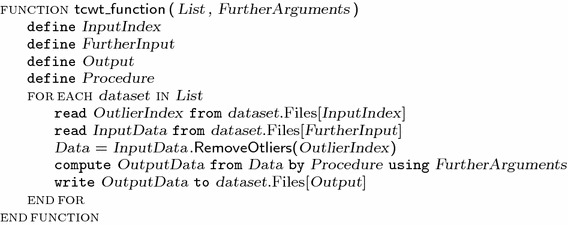

#### PCA and outlier detection

The multivariate outlier detection procedure proposed here is heavily based on PCA not only for computational reasons (due to small eigenvalues), but also because a multivariate outlier can strongly influence the dimensionality of the model, producing “fake” dimensions that survive PCA unless the outlier is excluded from the computation of the covariance matrix [23, p. 373].

An important distinction should be made at this point. PCA, as commonly used in ERP applications [26, 27] is performed in the time domain and it usually includes an additional rotation of the axes [28] following the initial one (4). This second rotation is aimed at obtaining more meaningful components, which, however, are *not* uncorrelated. In t-CWT, where PCA is only used in the pre/post-processing (see below), it does *not* include any additional rotations and the resulting components remain uncorrelated.

Consider an ERP represented via (28) in the frequency domain by the random vector $\tilde{\mathbf {{v}}}_{\mathbf {f}}$ and the single-trial sample $\tilde{\mathbf {{V}}}\!_{\mathbf {f}}$, the latter comprising two subsamples $\tilde{\mathbf {{V}}}\!_\mathbf {{f\,{\!\!_\mathcal {A}}}}$ and $\tilde{\mathbf {{V}}}\!_\mathbf {{f\,{\!_\mathcal {B}}}}$ corresponding to two different experimental conditions; $\tilde{\mathbf {{V}}}\!_\mathbf {{f\,{\!\!_\mathcal {A}}}}$ and $\tilde{\mathbf {{V}}}\!_\mathbf {{f\,{\!_\mathcal {B}}}}$ may in turn comprise subsamples of trials obtained from different individuals. PCT is performed according to (4), using the *total* covariance matrix obtained directly from $\tilde{\mathbf {{V}}}\!_{\mathbf {f}}$ (i.e. ignoring the subsample structure of $\tilde{\mathbf {{V}}}\!_{\mathbf {f}}$)

33 $$\begin{aligned} {\mathbf {v_p}} = \tilde{\mathbf {{v}}}_{\mathbf {f}}\,\mathbf {T_p},\quad \mathbf {V\!_p} =\tilde{\mathbf {{V}}}\!_{\mathbf {f}}\,\mathbf {T_p}. \end{aligned}$$

Then, components (represented by columns of $\mathbf {V\!_p}$ and $\mathbf {T_p}$) corresponding to eigenvalues smaller than a certain cutoff value (defined by one of the criteria mentioned above) are temporarily removed. The remaining $Q_\mathrm {p}$ variables $v_\mathrm {p}^i$, $i=1,2,\ldots Q_\mathrm {p}$, are normalized by (7). From the normalized variables $x^i$, for each *n*, the Mahalanobis distance $D_n$ from the *n*-th single-trial ERP ${\mathbf {x}}_n$ to the total mean $\mathbf {\overline{x}}$ is computed according to (8)

34 $$\begin{aligned} D_n^2 = D^2({\mathbf {x}}_n,\mathbf {\overline{x}}) = \sum _{i=1}^{Q_\mathrm {p}} \left( x^i_n-\overline{x}^i\right) ^2. \end{aligned}$$

With growing number of trials, each of the terms of the sum in (34) rapidly converges to the square of a standard normally distributed random variable. Hence, $D^2$ is approximately $\chi ^2$-distributed. With growing $Q_\mathrm {p}$, the square root of a $\chi ^2$-distributed random variable converges rapidly to a normal distribution as well, which means that $D=\sqrt{D^2}$ is approximately normally distributed.

The *n*th ERP trial is temporarily marked as an outlier if

35 $$\begin{aligned} D_n > \overline{D} + C\sigma \!_{\mathbf {{\tiny D}}}, \end{aligned}$$

where $\overline{D}$ is the mean, $\sigma \!_{\mathbf {\tiny D}}$ is the standard deviation of *D*, and *C* is a heuristically chosen coefficient, (usually $C\ge 2.5$).

The steps described above are repeated iteratively. Trials marked as outliers (represented by rows of $\mathbf {V\!_p}$) are excluded from the PCA and the computation of $\overline{D}$ and $\sigma \!_{\mathbf {\tiny D}}$ in the next iteration, but then they are tested again by (35) together with all other trials. Principal components are also excluded for one iteration only until their number remains unchanged through two consecutive iterations. After that, the PCA criterion is not applied any more and PCT is performed further with a fixed number of components. This is done in order to facilitate convergence. Also, in order to prevent oscillatory behavior, if the number of marked outliers does not increase after the current iteration, the outliers detected in the previous iteration are marked again together with those detected in the current iteration. The procedure ends when the set of detected outliers does not change any more (i.e. the same trials are marked in two consecutive iterations).

If $\tilde{\mathbf {{V}}}\!_{\mathbf {f}}$ comprises different individual datasets, a whole dataset is marked as an outlier (with all its trials) if a certain percentage of its trials are already marked. This criterion is applied, however, only if the number of single-trial outliers does not increase at the end of the current iteration. Note that, like single trials, whole data sets excluded at a certain step, can nevertheless be included again later.

As a result of the procedure described above, both rows of $\tilde{\mathbf {{V}}}\!_{\mathbf {f}}$ representing single-trial outliers and columns of $\mathbf {T\!_p}$ representing “noise components” or “outlier components” are deleted. The reduction of dimensionality is thus represented by the reduced PCT $\hat{\mathbf {{T}}}_{\mathbf {p}}$ and the corresponding “reduced” ERP matrices $\hat{\mathbf {{V}}}\!_{\mathbf {p}}$ and $\hat{\mathbf {{v}}}_{\mathbf {p}}$ where

36 $$\begin{aligned} \hat{\mathbf {{v}}}_{\mathbf {p}} = \tilde{\mathbf {{v}}}_{\mathbf {f}}\,\hat{\mathbf {{T}}}_ {\mathbf {p}},\quad \hat{\mathbf {{V}}}\!_{\mathbf {p}} =\tilde{\mathbf {{V}}}\!_{\mathbf {f}}\,\hat{\mathbf {{T}}}_{\mathbf {p}}. \end{aligned}$$

We use the “inverse” PCT $\hat{\mathbf {{T}}}_{\mathbf {p}}^{\mathsf {T}}$ to represent the dimensionality reduction in the frequency domain

37 $$\begin{aligned} \tilde{\mathbf {{v}}}_{\mathbf {f}}^{\mathbf {p}} = \hat{\mathbf {{v}}}_{\mathbf {p}}\,\hat{\mathbf {{T}}}_{\mathbf {p}}^{\mathsf {T}},\quad \tilde{\mathbf {{V}}}\!_{\mathbf {f}{}}^{^{\,\,\mathbf {p}}} = \hat{\mathbf {{V}}}\!_{\mathbf {p}}\,\hat{\mathbf {{T}}}_{\mathbf {p}}^{\mathsf {T}}. \end{aligned}$$

From (36) and (37), we finally obtain

38 $$\begin{aligned} \tilde{\mathbf {{v}}}_{\mathbf {f}}^{\mathbf {p}} = \tilde{\mathbf {{v}}}_{\mathbf {f}}\,\hat{\mathbf {{T}}}_{\mathbf {p}}\,\hat{\mathbf {{T}}}_ {\mathbf {p}}^{\mathsf {T}},\quad \tilde{\mathbf {{V}}}\!_{\mathbf {f}{}}^{^{\,\,\mathbf {p}}} = \tilde{\mathbf {{V}}}\!_{\mathbf {f}}\,\hat{\mathbf {{T}}}_{\mathbf {p}}\, \hat{\mathbf {{T}}}_{\mathbf {p}}^{\mathsf {T}}. \end{aligned}$$

Note that in (38) we assume that all rows of $\tilde{\mathbf {{V}}}\!_{\mathbf {f}}$ corresponding to outliers have already been deleted.

It is important to emphasize that, the principal components obtained by this procedure are identical with those which would be obtained if PCA were performed in the time domain using the filtered ERP, $\tilde{\mathbf {{v}}}$ (30). This is so, because the principal component axes that diagonalize the covariance matrix are unique (although their representation and the corresponding PCT depend on the representation of the input sample). The frequency domain representation is solely a matter of computational convenience due to dimensionality reduction by frequency filtering. Note also that although the dimensionality of the model is further reduced by the statistical “PCA filtering” (38), the dimensionality of the frequency domain representation remains unchanged.

The above procedure can be additionally applied to each of the two subsamples $\tilde{\mathbf {{V}}}\!_{\mathbf {f\,{\!\!_\mathcal {A}}}}$ and $\tilde{\mathbf {{V}}}\!_{\mathbf {f\,{\!_\mathcal {B}}}}$ separately, using the principal components obtained from the whole sample $\tilde{\mathbf {{V}}}\!_{\mathbf {f}}$. The only difference is that no PCA is done any more (because the components have already been fixed).

**Fig. 2:**
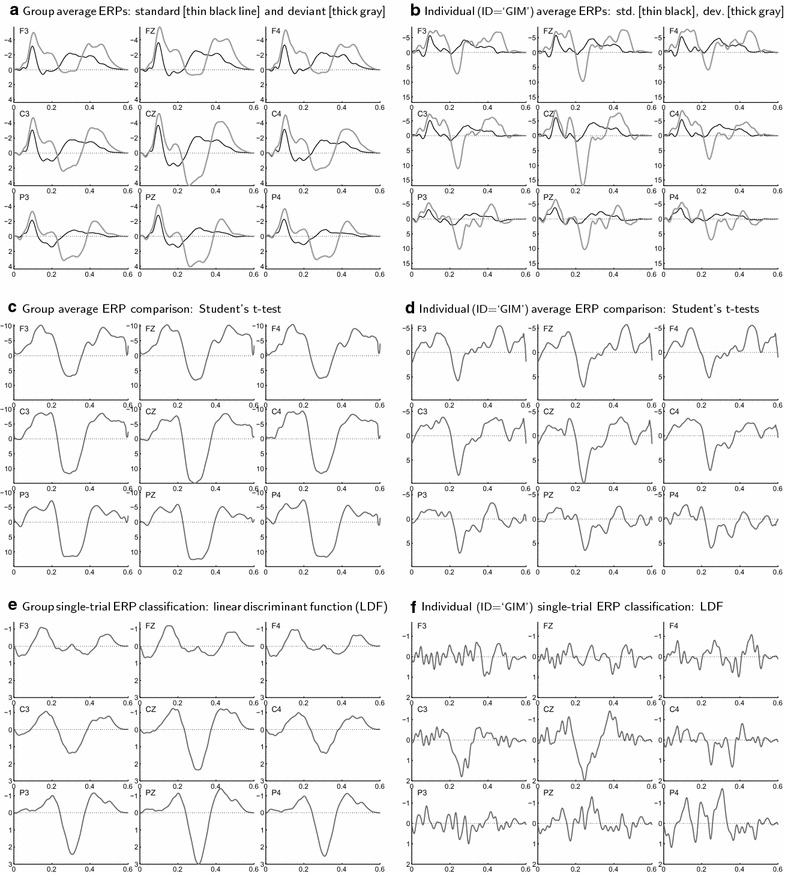
Group and individual ERP averages, Student’s t-value curves, and LDFs. *Upper plots* **a** grand average and **b** individual average ERPs (in microvolts, as functions of time in seconds) elicited by 242 standard (*thin black line*) and 38 deviant (*thick gray line*) stimuli in a passive oddball paradigm. The individual dataset was obtained from the EEG of participant ‘GIM’ (see Table 3 for LDA classification results). The ERPs were filtered by a low-pass filter with cutoff frequency 25 Hz ($\textit{S}_{\textit{c}}$ = 40 ms) and by a statistical PCA filter with proportion of explained variance $\textit{P}\!_{\textit{v}}$ = 99 %. The *middle plots* **c** and **d** show the corresponding ERP difference t-value curves (without any correction for multiple comparisons), while the *lower plots* **e** and **f** show the normalized linear discriminant functions (LDFs) obtained by the t-CWT method for the whole group and for the individual dataset. The LDFs were computed for $\textit{S}_{\textit{c}}$ = 40 ms, $\textit{P}\!_{\textit{v}}$ = 99 %, and log-grid sampling rate $\textit{R}$ = 15 pps

It is important to note that the pre-processing procedures described above can be used independently from the t-CWT feature extraction (described below). For instance, the ERP sample $\tilde{\mathbf {{V}}}_{\mathbf {f}}^{\mathbf {p}}$ (filtered and free from outliers) can be represented back in the time domain by (26):

39 $$\begin{aligned} \tilde{\mathbf {{V}}}^{\mathbf {p}} = \tilde{\mathbf {{V}}}_{\!\mathbf {f}}^{\mathbf {p}}\hat{\mathbf {{T}}}_{\mathbf {f}}^{\mathsf {T}}, \end{aligned}$$

and then used as input for other assessment procedures. In this article, the representation (39) is used solely for visualization purposes (see Fig. 2).

In the t-CWT software [16, 17], the PCA-based multivariate outlier detection procedure (33–38) is implemented by the function tcwt_f2pc; outlier detection for each experimental condition separately with fixed PCT obtained by tcwt_f2pc is implemented by the function tcwt_pc2cnd2ri (Table 1).

### t-CWT

#### Log-grid sampling

In (18), the number of rows of the CWT matrix $\mathbf {T_w}$ is equal to the number of components of $\mathbf {w}$, which is equal to the number of sampling points in the *s*-*t*-plane of the wavelet $\psi \big ((\tau -t)/s\big )$ in (16). This number can be significantly reduced by using the log-grid introduced in [7] instead of a regular sampling grid. The vertices $(s^g,t^{g,h})$ of the log-grid (Fig. 1b) are defined by

40 $$\begin{aligned} s^g = S_0\exp \!\bigg (\!\ln (2)\frac{g}{R}\bigg ), \quad t^{g,h} = s^g\frac{h}{R}, \end{aligned}$$

where $S_0$ is some unit scale, *R* is the *scale-invariant* sampling rate measured in points per scale (pps), and *g* and *h* take integer values (including negative and zero). The scale invariance can be expressed by the two properties: $s^{g+R}=2s^g$ (i.e., we have *R* grid lines per octave), and $t^{g,h+R}=t^{g,h}+s^g$ (i.e., on each grid line, we have *R* sampling points per time interval of length $s^g$). The special case $R=1$ yields the DWT with its dyadic structure [8, 21]. The t-CWT application uses only the part of the infinite log-grid (40) confined by the rectangle

41 $$\begin{aligned} \frac{S_c}{2} \le s^g \le 4T,\quad 0 \le t^{g,h} \le T. \end{aligned}$$

In (41), the minimal scale $S_c/2$ corresponds to the maximal frequency $2f_c$ in (24). For a large number of log-grid vertices $N\!_G$, a good approximation is given by

42 $$\begin{aligned} N\!_G\approx 3 R^2\frac{T}{S_c}. \end{aligned}$$

Thus, the number of CWT sampling points is significantly reduced, compared to the number of vertices of a rectilinear grid with a regular spacing defined in the same rectangle (41), e.g., by the original sampling frequency $R_0$ applied to both axes as in (1). Note that the sampling frequency *R* of the log-grid can be chosen to correspond to the original sampling rate $R_0$ by setting $R\!=\!S_c R_0$, but this is not necessary and *R* can as well take an independent value $R\!\ne \!S_c R_0$.

In the t-CWT software [16, 17], the log-grid $(s^g,t^{g,h})$ (40–41) is implemented by a matrix of vertices computed by the function tcwt_prm2mat (Table 1).

#### CWT and t-CWT from the frequency domain

As in (18), we define the CWT ${\hat{\mathbf {T}}}_{\mathbf {w}}$ of the filtered ERP defined by (26) as

43 $$\begin{aligned} \hat{\mathbf {{w}}} = \hat{\mathbf {{v}}}\hat{\mathbf {{T}}}_{\mathbf {w}},\quad \! \hat{\mathbf {{W}}} =\hat{\mathbf {{V}}}\hat{\mathbf {{T}}}_{\mathbf {w}}, \end{aligned}$$

Substituting (26) in (43), we obtain

44 $$\begin{aligned} \hat{\mathbf {{w}}} = \hat{\mathbf {{v}}}_{\mathbf {f}}\,\tilde{\mathbf {{T}}}_{\mathbf {w}},\quad \! \hat{\mathbf {{W}}} =\hat{\mathbf {V}}\!_{\mathbf {f}}\,\tilde{\mathbf {T}}_{\mathbf {w}}, \end{aligned}$$

where

45 $$\begin{aligned} \tilde{\mathbf {T}}_{\mathbf {w}} = \hat{\mathbf {{T}}}_{\mathbf {f}}^{\mathsf {T}}\,\hat{\mathbf {{T}}}_{\mathbf {w}}. \end{aligned}$$

The rows of the inverse DFT $\mathbf {T_f^\mathsf {T}}$ are the discrete vector representations of the basic EEG oscillations $\sin (2\pi f^j t)$ and $\cos (2\pi f^j t)$ with frequencies $f^j\!=\!j/T$, where $j=0,1,2...$ such that $f^j\le 2f_c$ according to (24). Hence, $\tilde{\mathbf {{T}}}_{\mathbf {w}}$ is computed by (16) using the log-grid sampling (40–42). The convolution integrals are represented by the corresponding sums with the original sampling rate $R_0$.

Like $\mathbf {T_w}$ (18), $\tilde{\mathbf {{T}}}_{\mathbf {w}}$ is a block diagonal matrix built from *K* identical blocks (one per channel). The size of each block is $N\!_F\!\times \!N\!_G$ where $N\!_F$ and $N\!_G$ are the number of frequency components (31, 32) and the number of log-grid vertices (42), respectively. From (32) and (42) we obtain the following approximation:

46 $$\begin{aligned} N\!_F N\!_G\approx 12 R^2 \frac{T^2}{S_c^2}. \end{aligned}$$

The current implementation of t-CWT uses a Mexican Hat wavelet defined by

47 $$\begin{aligned} \psi (t) = ( 1 - 16 t^2 ) e^{-8t^2}. \end{aligned}$$

Note that (47) differs from the standard definition of the Mexican Hat, $\psi (t)=(1-t^2)\exp (-t^2/2)$. The unity scale defined by (47) is four times larger than the standard. This is done for convenience: defined in this way, the scale corresponds better to the durations of the ERP waves matched by the wavelet and to the periods of the oscillations $\sin (2\pi f^j t)$ and $\cos (2\pi f^j t)$ in (45).

In order to exclude the outliers detected above, we apply the obtained CWT $\tilde{\mathbf {{T}}}_{\mathbf {w}}$ to the “reduced” matrices ${\tilde{\mathbf {v}}}_{\mathbf {f}}^{{\mathbf {p}}}$ and $\tilde{\mathbf {V}}_{\mathbf {f}}^{{\mathbf {p}}}$ defined by (38)

48 $$\begin{aligned} {\tilde{\mathbf {w}}} = \tilde{\mathbf {v}}_{\mathbf {f}}^{{}_{\mathbf {p}}}\, \tilde{\mathbf {T}}_{\mathbf {w}},\quad {\tilde{\mathbf {W}}} =\tilde{\mathbf {V}}_{\mathbf {f}}^{{\mathbf {p}}}\, \tilde{\mathbf {T}}_{\mathbf {w}}. \end{aligned}$$

The t-CWT features are computed by (19, 20) and the t-CWT matrix $\tilde{\mathbf {T}}^\star _{\mathbf {w}}$ is obtained from $\tilde{\mathbf {T}}_{\mathbf {w}}$ by retaining only the columns that represent the t-CWT features (20). Substituting $\tilde{\mathbf {T}}_{\mathbf {w}}$ in (48) with $\tilde{\mathbf {T}}^\star _{\mathbf {w}}$, we obtain

49 $$\begin{aligned} \mathbf {w^\star } = \tilde{\mathbf {v}}_{\mathbf {f}}^{{\mathbf {p}}}\,\tilde{\mathbf {T}}^\star _{\mathbf {w}},\quad \mathbf {W^\star } = \tilde{\mathbf {V}}_{\mathbf {f}}^{{\mathbf {p}}}\,\tilde{\mathbf {T}}^\star _{\mathbf {w}}. \end{aligned}$$

In the t-CWT software [16, 17], the computation of the CWT matrix $\tilde{\mathbf {{T}}}_{\mathbf {w}}$ (16–18, 43–45) is implemented by the function tcwt_prm2mat; the computations of the t-CWT scalogram $\mathtt {t}^k(s,t)$ (19), the t-CWT extrema and the t-CWT matrix $\tilde{\mathbf {{T}}}^\star _{\mathbf {w}}$ (49) are implemented by the functions tcwt_f2cwss and tcwt_f2x (Table 1).

### Post-processing in the feature space

The t-CWT features $\mathbf {w^\star }$ are still strongly correlated, because one and the same ERP component is found in more than one EEG channel represented by at least one extremum in each channel’s sub-scalogram. Furthermore, not all such sets of extrema represent significant ERP components. For these reasons, the dimensionality of the feature space is reduced further by PCA and step-down selection of principal components. Finally, the LDF is computed in the reduced feature space.

#### PCA and step-down test

PCT is performed in the feature space according to (4)

50 $$\begin{aligned} \mathbf {w^\star _p} = \mathbf {w^\star }\,\mathbf {T^\star _p},\quad \mathbf {W^\star _p} = \mathbf {W^\star }\,\mathbf {T^\star _p}, \end{aligned}$$

and the set of components is reduced according to one of the PCA criteria mentioned above. Then, a subset of components, “selected principal components” (SPC), is selected by a step-down test [23, pp. 111, 177, 217] based on the natural ordering of the components (sorted by eigenvalue). The “reduced” matrices ${\hat{\mathbf {T}}}^{\mathbf {\star }}_{\mathbf {p}}$, ${\hat{\mathbf {W}}}^ {\mathbf {\star }}_{\mathbf {p}}$ and ${\hat{\mathbf {w}}}^{\mathbf {\star }}_{\mathbf {p}}$ are obtained by deleting the columns corresponding to the eliminated components in $\mathbf {T^\star _p}$, $\mathbf {W^\star _p}$ and $\mathbf {w^\star _p}$, respectively:

51 $$\begin{aligned} {\hat{\mathbf {w}}}^{\mathbf {\star }}_{\mathbf {p}} = \mathbf {w^\star }\,{\hat{\mathbf {T}}}^{\mathbf {\star }}_{\mathbf {p}},\quad {\hat{\mathbf {W}}^{\mathbf {\star }}_{\mathbf {p}}} = \mathbf {W^\star }\,{\hat{\mathbf {T}}^{\mathbf {\star }}_{\mathbf {p}}}, \end{aligned}$$

#### LDA

The LDF $\mathbf {d_w^\star }$ is computed in the reduced feature space as in (11):

52 $$\begin{aligned} \mathbf {d_w^\star } = {\hat{\mathbf {S}}^{\mathbf {\star -1}}_{{\mathbf {p}}\,{\!\!_\mathcal {A}}{\!_\mathcal {B}}}}\, \left( {\hat{\mathbf {\overline{w}}}}^{\mathbf {\star }}_{{\mathbf {p}}\,{\!\!_\mathcal {A}}} - {\hat{\mathbf {\overline{w}}}^{\mathbf {\star }}}_{{\mathbf {p}}\,{\!_\mathcal {B}}} \right) ^\mathsf {\!T}, \end{aligned}$$

where ${\hat{\mathbf {\overline{w}}}^{\mathbf {\star }}_{\mathbf {p\,{\!\!_\mathcal {A}}}}}$ and ${\hat{\mathbf {\overline{w}}}^{\mathbf {\star }}_{\mathbf {p\,{\!_\mathcal {B}}}}}$ are the respective means of the two subsamples ${\hat{\mathbf {W}}^{\mathbf {\star }}_{\mathbf {{p\,{\!\!_\mathcal {A}}}}}}$ and ${\hat{\mathbf {W}}^{\mathbf {\star }}_{{\mathbf {p}}\,{\!_\mathcal {B}}}}$, and ${\hat{\mathbf {S}}^{\mathbf {\star }}_{{\mathbf {p}}\,{\!\!_\mathcal {A}}{\!_\mathcal {B}}}}$ is the pooled covariance matrix. The LDA separation plane is defined as in (13–15) by

53 $$\begin{aligned} {\hat{\mathbf {w}}}^{\mathbf {\star }}_{\mathbf {p}}\,\mathbf {d_w^{\star }} = \tfrac{1}{2}\left( {\hat{\mathbf {\overline{w}}}}^{\mathbf {\star }}_{{\mathbf {p}}\,{\!\!_\mathcal {A}}} + {\hat{\mathbf {\overline{w}}}^{\mathbf {\star }}_{{\mathbf {p}}\,{\!_\mathcal {B}}}} \right) \mathbf {d_w^{\star }}+\ln \left( \frac{p_{\!_\mathcal {B}}}{p_{\!\!_\mathcal {A}}}\right) . \end{aligned}$$

Now, the transformations (28), (38), (49), and (51) can be chained together in order to represent the LDF $\mathbf {d_w^\star }$ and the LDF value ${\hat{\mathbf {w}}^{\mathbf {\star }}_{\mathbf {p}}\,\mathbf {d_w^\star }}$ back in the frequency domain and in the time domain:

54 $$\begin{aligned} {\hat{\mathbf {w}}}^{\mathbf {\star }}_{\mathbf {p}}\,\mathbf {d_w^\star } = \mathbf {v^{}_f}\,\mathbf {d_f^\star } = \mathbf {v}\mathbf {d^\star }, \end{aligned}$$

where

55 $$\begin{aligned} \mathbf {d_f^\star } = {\hat{\mathbf {T}}}_{\mathbf {p}}\, {\hat{\mathbf {T}}_{\mathbf {p}}^\mathsf {T}}\, \tilde{\mathbf {T}}^\star _{\mathbf {w}}\, {\hat{\mathbf {T}}}^{\mathbf {\star }}_{\mathbf {p}}\,\mathbf {d_w^\star }, \end{aligned}$$

and

56 $$\begin{aligned} \mathbf {d^\star } = \tilde{\mathbf {T}}_{\mathbf {f}}\,\mathbf {d_f^\star }. \end{aligned}$$

Finally, it might be useful to mention also the continuous time domain representation of the LDF value:

57 $$\begin{aligned} \mathbf {v}\mathbf {d^\star } = \sum _{k=1}^K \int \limits _0^T\,v^k(t)\,d^{\star k}(t)\,dt, \end{aligned}$$

where *K* is the number of channels, *T* is the length of the time window, and $v^k(t)$ and $d^{\star k}(t)$ are the continuous representations of $\mathbf {v}$ and $\mathbf {d^\star }$ respectively (see Fig. 2).

In the t-CWT software [16, 17], the PCA-based step-down reduction of the t-CWT features (50–51) and the computation of the LDF $\mathbf {d_w^\star }$ (52) and $\mathbf {d_f^\star }$ (55) are implemented by the function tcwt_x2ld (Table 1).

### Algorithm summary with links to the t-CWT software

In Table 1, the processing steps delineated above are summarized and linked to the corresponding functions and input/output data files defined by the t-CWT software [16] (for a detailed description of these files and functions, see the t-CWT software documentation [17]). Output file names displayed in the last column of Table 1 are provided with references to corresponding mathematical variables and equations defined in this article. The wildcard symbol “ * ” denotes an ERP dataset name and indicates that the corresponding t-CWT functions accept a whole list of such names as an argument and then iterate over the list, thus processing multiple ERP datasets in a single call (see the pseudocode at the bottom of Table 1).

### Computational demands

The most computationally demanding procedures are the PCA and the t-CWT including CWT and extremum detection from a scalogram.

#### PCA

The number of matrix elements of the PCT is $K^2 N\!_F^{\,2}$. The covariance matrix has the same number of elements. Using (32) we obtain the approximate total number of elements of both matrices:

58 $$\begin{aligned} N\!_P\approx 32 K^2 \frac{T^2}{S_c^2}. \end{aligned}$$

Both the memory and the processing time required for PCA are approximately proportional to $N\!_P$. Each double-precision matrix element needs eight bytes of memory. The processing time depends on the central processing unit (CPU), but, as a rule of thumb, one microsecond per matrix element can be used for rough estimation of the time needed for one iteration of the PCA-based multivariate outlier detection procedure (see above).

#### t-CWT

The approximate number of (non-zero) CWT matrix elements per channel is given by (46). The amount of memory consumed by t-CWT is proportional only to this number and does not depend on the number of channels *K*, because the t-CWT application [16, 17] performs CWT on one channel at a time. Furthermore, if the processed dataset is too big, t-CWT does not transform all trials at once, but processes smaller blocks of trials (the block size is controlled by an input parameter), thus limiting the memory demand.

The time t-CWT needs to process one scalogram (comprising *K* sub-scalograms) is approximately proportional to the total number of non-zero CWT matrix elements $N\!_W\!=\!K N\!_F N\!_G$. From (46) we obtain the following approximation:

59 $$\begin{aligned} N\!_W\approx 12 K R^2 \frac{T^2}{S_c^2} . \end{aligned}$$

Again, as a rule of thumb, one microsecond per matrix element can be used for rough estimation of the time t-CWT needs to process one scalogram (with *K* channels).

The t-CWT software package [16, 17] includes the function tcwt_prm2info that evaluates both the exact values of $N\!_P$ and $N\!_W$ and their approximations computed by (58, 59) for a given set of input parameters. Further, tcwt_prm2info makes a rough estimate of the computational demands based on the simple assumption of eight bytes of working memory and one microsecond of processing time per matrix element. All this is done in real time, without actually creating the corresponding matrices and can be very useful in the planning stage of a t-CWT project when the available computational resources must be taken into account.

#### Example

Consider, e.g., the following settings: *K* = 64, *T* = 1 s, $S_c$ = 50 ms ($f_c$ = 20 Hz), and *R* = 15 pps. Then we would have the following (exact) numbers obtained with tcwt_prm2info: $N\!_F$ = 81, $N\!_G$ = 13,276, $N\!_P$ = 53,747,712, and $N\!_W$ = 68,822,784. The memory used for PCA would be about 430 MB and for CWT (transforming 1,000 trials at ones), about 116 MB. The estimated processing time for one PCA iteration would be about 54 s, and for one CWT scalogram, about 69 s. On a powerful hardware (see below), however, these computational times can be significantly shorter (see Table 2).

### Example: oddball paradigm

In this section, the t-CWT is demonstrated on example ERP data [18] obtained in a passive oddball paradigm [19, 20]. Since these datasets have already been described in detail elsewhere [11], only the most important information about the experiment is provided here.

#### Datasets

ERP datasets were obtained from 36 healthy participants (right-handed, mean age = 27 years, 20 females) in a passive oddball task [19, 20], in which 255 standard and 45 deviant stimuli were presented at a constant rate in a randomized sequence. The standard and the deviant stimuli were 50-ms-long, 75-dB-loud sine tones, with a frequency of 1.3 and 0.8 kHz, respectively; the interstimulus interval was 0.8 s. The participants were instructed just to listen attentively to all tones (passive task). Digitized EEG [time resolution: 2 ms/step (500 Hz), voltage resolution: 0.1678 microvolts/step] was continuously recorded from nine scalp positions according to the 10–20 system: Fz, Cz, Pz, F3, F4, C3, C4, P3, and P4. All electrodes were referenced to the linked mastoids. Electrical eye activity was recorded by bipolar acquisition from the following sites: the two lateral orbital rims for horizontal eye movements, and FP2 and a site below the right eye for vertical eye movements and eye blinks. The first nine datasets (in alphabetical order) were excluded for technical reasons (in order to reduce the whole dataset archive to 100 MB for online storage and download purposes). The remaining 27 datasets were processed with t-CWT.

**Table 2:** Individual hold-out error rates (average %) and computational demands

${\mathbf {S}}_{{\mathbf {c}}}$ (**ms**)	${{\mathbf {f}}}_{{\mathbf {c}}}$ **(Hz)**	**R** **(pps)**	**PCA**	**Average holdout error rates (%)**						**Freq.**	**Log**	**Matrix**	**ME ratio:**	**Processing time**	
			${{\mathbf {P}}}_{{\mathbf {v}}}$ **(%)**	**A priori: 50 %**			**A priori: 13.6 %**			**Dom.**	**Grid**	**Elements**	$\underline{{\mathbf {N}}_{{\mathbf {F}}} {\mathbf {N}}_{{\mathbf {G}}} {\mathbf {S}}_{{\mathbf {c}}}^{{\mathbf {2}}}}$	**Total** (**h:min**)	**Per ME** (**ns**)
				**Std**	**Dev**	**Tot**	**Std**	**Dev**	**Tot**	${\mathbf {N}}_{{\mathbf {F}}}$	${\mathbf {N}}_{{\mathbf {G}}}$	${\mathbf {K}} {\mathbf {N}}_{{\mathbf {F}}}$ ${\mathbf {N}}_{{\mathbf {G}}}$	${\mathbf {R}}^{{\mathbf {2}}}$ ${\mathbf {T}}^{{\mathbf {2}}}$		
250	4	10	95	22.0	31.7	23.3	2.2	66.8	11.0	9	774	62,694	12.1	0:07	858
100	10	15	95	19.0	32.8	20.9	2.7	61.9	10.7	25	4,339	976,275	13.4	0:57	467
100	10	15	97	18.2	32.2	20.1	2.9	60.6	10.8	25	4,339	976,275	13.4	0:58	474
100	10	15	99	16.9	32.0	18.9	2.8	57.6	10.2	25	4,339	976,275	13.4	0:59	476
50	20	15	95	18.7	32.9	20.7	2.9	61.3	10.8	49	8,320	3,669,120	12.6	3:02	394
50	20	15	97	17.5	33.2	19.7	2.8	58.3	10.3	49	8,320	3,669,120	12.6	3:04	398
50	20	15	99	15.4	33.7	17.9	2.9	55.5	10.0	49	8,320	3,669,120	12.6	3:10	412
40	25	15	95	17.8	32.7	19.8	2.7	61.4	10.6	61	10,341	5,677,209	12.5	4:41	393
40	25	15	97	16.9	32.6	19.0	2.8	58.8	10.4	61	10,341	5,677,209	12.5	4:37	388
40	25	15	99	15.3	33.2	17.7	3.0	55.0	10.0	61	10,341	5,677,209	12.5	4:44	397
40	25	20	99	15.4	31.8	17.6	3.0	55.2	10.1	61	18,257	10,023,093	12.4	8:19	395
40	25	25	99	15.0	32.7	17.4	2.7	55.7	9.9	61	28,245	15,506,505	12.3	13:41	420
30	33	15	99	15.5	34.5	18.1	3.3	56.5	10.5	81	13,657	9,955,953	12.3	7:46	372

#### Data processing

Before feeding the data into the t-CWT processor, the raw EEG curves were segmented and corrected for eye blink and eye movement artifacts, by a standard procedure [29, 30]. The epoch length was 1 s, starting 100 ms before stimulus onset. Then, the datasets were checked for series of more than one deviant trials. Only the first trials of such series were retained, the following deviant trials as well as the first subsequent standard trial were deleted. The first ten trials of each dataset were also deleted. As a result of this reduction, in each dataset, remained 242 standard trials and 38 deviant trials. Thus, the *a priori* probabilities (15, 53) were $p_s=86.4$ % for standard trials and $p_d=13.6$ % for deviant trials. The datasets were converted from ASCII format to the internal t-CWT format by the function tcwt_ascii2tmat [16, 17] and the epochs were reduced to windows of interest starting at stimulus onset and ending 600 ms later. The signals in these windows were then referenced to the 100 ms pre-stimulus baseline. These signals were processed by t-CWT.

In its current implementation [16, 17], t-CWT starts with a call to the function tcwt_prm2info which gives a rough estimate of the memory demands and the computational time (see above). The next call is to the function tcwt_prm2mat that creates and saves to a file those transformation matrices which do not depend on the data, but are functions of global input parameters only (see Table 1). These matrices are the DFT $\tilde{\mathbf {T}}_{\mathbf {f}}$ (29) and the CWT $\tilde{\mathbf {T}}_{\mathbf {w}}$ (45), and they depend on the original sampling rate $R_0$ (1), the length of the time window *T* (1), the fade-in and the fade-out times $T_\mathrm {in}$ and $T_\mathrm {out}$ of the window function (27), the log-grid sampling rate *R* (40), and the cutoff scale $S_c$ (22). While $R_0$ is defined by the time resolution setting of the EEG amplifier (in this case, $R_0$ = 500 Hz), the other parameters can be varied to achieve best LDA classification results. In the current example, *T*, $T_\mathrm {in}$ and $T_\mathrm {out}$ were kept fixed: *T* = 600 ms, $T_\mathrm {in}$ = 20 ms, $T_\mathrm {out}$ = 200 ms, while *R* and $S_c$ took the following values: $R=$ 10, 15, 20, and 25 pps; $S_c=$ 250, 100, 50, 40, and 30 ms. Note that these values of $S_c$ correspond to cutoff frequencies $f_c=$ 4, 10, 20, 25, and 33.3 Hz, respectively (22).

After computing $\tilde{\mathbf {T}}_{\mathbf {f}}$ and ${\tilde{\mathbf {T}}}_{\mathbf {w}}$, the data were represented in the frequency domain by (29) using the function tcwt_t2f (Table 1). The outlier rejection procedure described above (33–38) was performed on each dataset, first on the whole dataset (by the function tcwt_f2pc), then on each of the two subsets, standard and deviant (by the function tcwt_pc2cnd2ri, see Table 1). The greatest eigenvalues explaining a certain percentage $P\!_v$ of the variance were retained after PCT. Different values of $P\!_v$ were tried in order to minimize the LDA classification error rates (see below): $P\!_v=$ 95, 97, and 99 %. The outlier criterion was defined by setting $C=2.7$ in (35). (Other values of *C* ranging from 2.5 to 9.9 were tried as well, but without any substantial effect on the results; see the Limitations section in the “Discussion” section.)

As a next step, the t-CWT features $\mathbf {w^\star }$ were computed (for each dataset) as described in (43–49) using the function tcwt_f2x (Table 1). The obtained set of t-CWT features was reduced further by PCAs (50) and step-down tests (51), implemented by the function tcwt_x2ld (Table 1). The same PCA criterion with the same value of $P\!_v$ as in the outlier rejection procedure was applied. The overall $\alpha$-level for the step-down test was set to $\alpha _\text {sd}=0.3$. Finally, the LDFs $\mathbf {d^\star }$ of the individual datasets were obtained by (52), also implemented by the function tcwt_x2ld (Table 1).

LDA classification was performed according to (13–15, 53) and classification error rates were computed using the hold-out method and the split-half method [23, p. 244] for both unknown (i.e., equal: $p_s\!=\!p_d$ = 50 %) and known (i.e., oddball: $p_s$ = 86.4 %, $p_d$ = 13.6 %, see above) *a priori* probabilities.

In the “individual hold-out” method, all of the above steps but the first one (outlier rejection from the whole dataset) were performed on a dataset obtained from the original one by excluding one single trial. The LDF thus obtained was used to classify the excluded trial as standard or deviant by (14) and (15). The error rates were obtained by repeating the procedure for each single trial and each dataset and comparing the result with the true category of each trial (standard or deviant). The hold-out method is very efficient, because it is almost unbiased and it uses the whole available statistical power, but it is also a computationally demanding procedure. In the t-CWT software [16, 17], the individual hold-out method is implemented by the function tcwt_f2holdout.

In the “individual split-half” method, all steps were performed on the first half of the trials of each dataset (extracted by the function tcwt_f2split [16, 17]). The LDFs obtained from these “training datasets” were then applied to the second halves of the datasets, the “test datasets”. This method is quick and simple, but it has considerable loss of statistical power as a major disadvantage.

“Individual biased” error rates were also computed in order to demonstrate the bias resulting from using the same dataset (without excluding any trials) for both training and testing.

In order to demonstrate multivariate hypothesis testing, only the last 9 deviant trials and 50 standard trials of each dataset were used as training dataset, while the first half of the trials were used as test dataset. The SPCs obtained from each training dataset by the step-down method were applied to each test dataset and subjected to Hotelling’s T${}^2$-test. Both the individual split-half error rates and the biased error rates, as well as Hotelling’s T${}^2$-tests were performed with the function tcwt_f2stats [16, 17].

Finally, all individual datasets were pooled together into one large group dataset (using the function tcwt_f2pool [16, 17]). Outlier rejection (using tcwt_f2pc and tcwt_pc2cnd2ri) was performed with the average eigenvalue as a PCA criterion in order to emphasize group features and to suppress individual and/or oscillatory features. The single-trial outlier criterion was defined by setting $C=2.5$ in (35). (Again, experimenting with other values of *C* had virtually no effect on the results; see the Limitations section in the “Discussion” section.) The dataset outlier criterion was defined by setting the minimum number of trials retained to 50 % of the trials in the dataset. t-CWT, PCA (again, with the average eigenvalue criterion), step-down test, and LDA were performed as above (using tcwt_f2x and tcwt_x2ld). A variation of the hold-out method, the “group hold-out” was used to obtain classification error rates for the individual datasets: instead of excluding one single trial at a time, one whole individual dataset was excluded at each iteration. Each error rate obtained in this way is based on a group LDF applied to the respective excluded dataset.

The t-CWT software [16, 17], provides the function tcwt_ri2ri1out that creates systematically hold-out indices of the pooled group dataset which are specially designed for the implementation of the group hold-out method. In a group hold-out index corresponding to a given individual dataset, all single trials belonging to this dataset are marked as “outliers”. The functions tcwt_pc2cnd2ri, tcwt_f2x, tcwt_x2ld, and tcwt_f2stats can use these index files to compute the corresponding group hold-out t-CWT features, group hold-out LDFs, and group hold-out error rates. The hold-out mode of operation of these functions is very similar to their normal mode outlined in the pseudocode at the bottom of Table 1 (i.e., they iterate through the list of individual datasets, loading in each iteration the corresponding group hold-out index file and other group hold-out input files, performing the respective transformations and computations, and saving the results in corresponding group hold-out output files).

For all obtained error rates (individual hold-out, individual split-half, individual biased, and group hold-out), binomial distribution *p*-values were computed (by tcwt_f2stats and tcwt_f2holdout) to test the hypotheses whether these error rates were better (i.e., smaller) than the chance classification error rates defined by the *a priori* probabilities $p_d=50$ % or $p_d=13.6$ % (see above).

**Fig. 3:**
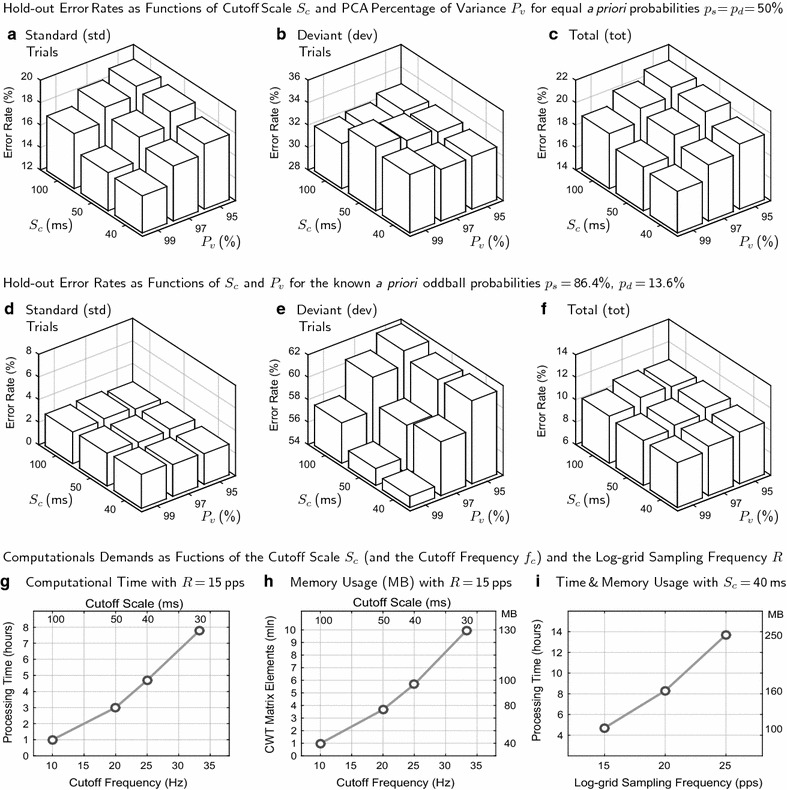
Hold-out error rates and computational demands as functions of $\textit{S}_{\textit{c}}$, $\textit{P}\!_{\textit{v}}$ and $\textit{R}$. These plots visualize the most important results displayed in Table 2. Plots **a**–**c** show the average classification errors obtained with the individual hold-out method with equal *a priori* probabilities $\textit{p}_{\textit{s}}\!=\!\textit{p}_{\textit{d}}$ =50 % (14) for different values of the cutoff scale $\textit{S}_{\textit{c}}$ and the percentage of variance $\textit{P}\!_{\textit{v}}$ explained by PCA. The corresponding error rates obtained by using the knowledge of the real oddball probabilities $p_s$ = 86.4 % and $\textit{p}_{\textit{d}}$ = 13.6 % (15) are displayed in the plots **d**–**f**. The approximate processing time as a function of $\textit{S}_{\textit{c}}$, or the respective cutoff frequency $\textit{f}_{\textit{c}}$ (22), is displayed in plot **g**. Plot **h** shows how the number of the non-zero CWT matrix elements, measured in millions (mln), and the respective memory usage, measured in megabytes (MB), depend on ${\textit{S}}_{\textit{c}}$ or $\textit{f}_{\textit{c}}$. Plot **i** shows how both processing time and memory usage increase as a function of the log-grid sampling rate *R* (40)

**Table 3:** Apparent error rates (%) and Hotelling tests for individual datasets

**No**.	**ID**	**Group hold-out**					**Individual biased**					**Individual split-half**					**Ind. hold-out**			**Hotelling**
		${\mathbf {N}}_{{\mathbf {X}}}$	${\mathbf {N}}_{{\mathbf {P}}}$	**Std**	**Dev**	**Tot**	${\mathbf {N}}_{{\mathbf {X}}}$	${\mathbf {N}}_{{\mathbf {P}}}$	**Std**	**Dev**	**Tot**	${\mathbf {N}}_{{\mathbf {X}}}$	${\mathbf {N}}_{{\mathbf {P}}}$	**Std**	**Dev**	**Tot**	**Std**	**Dev**	**Tot**	${\mathbf {p}}$-**value**
1.	GIM	127	6	0.4	76.3	10.7	120	7	2.1	21.1	$4.6^{*}$	131	5	1.7	15.8	$3.6^{*}$	2.9	23.7	$5.7^{*}$	0.0000$^{*}$
2.	GOI	137	6	8.3	55.3	14.6	118	5	2.5	44.7	$8.2^{*}$	129	4	3.3	42.1	$8.6^{*}$	1.7	47.4	$7.9^{*}$	0.0001$^{*}$
3.	GUS	129	6	0.0	100.0	13.6	159	8	2.9	44.7	$8.6^{*}$	126	4	4.1	73.7	13.6	4.5	57.9	11.8	0.0109$^{*}$
4.	HAH	128	6	0.8	86.8	12.5	140	4	2.1	63.2	10.4	129	4	7.4	68.4	15.7	3.7	71.1	12.9	0.0011$^{*}$
5.	HEA	132	6	0.8	89.5	12.9	130	5	2.9	68.4	11.8	141	2	8.3	47.4	13.6	4.1	68.4	12.9	0.0004$^{*}$
6.	HII	134	6	0.8	94.7	13.6	135	8	2.9	39.5	$7.9^{*}$	138	7	5.8	73.7	15.0	5.8	57.9	12.9	0.0919
7.	JUE	135	6	5.0	97.4	17.5	139	4	2.1	81.6	12.9	155	3	3.3	94.7	15.7	2.5	86.8	13.9	0.7760
8.	KAA	135	6	0.8	57.9	$8.6^{*}$	181	5	0.8	31.6	$5.0^{*}$	185	2	0.8	52.6	$7.9^{*}$	0.8	36.8	$5.7^{*}$	0.0000$^{*}$
9.	KAC	129	6	9.1	71.1	17.5	137	8	3.7	55.3	10.7	148	5	2.5	78.9	12.9	3.7	65.8	12.1	0.1185
10.	KUD	127	6	0.8	86.8	12.5	139	4	2.9	57.9	10.4	131	1	0.8	68.4	10.0	2.9	71.1	12.1	0.0572
11.	MAH	128	6	2.5	71.1	11.8	131	2	3.7	55.3	10.7	136	3	0.8	84.2	12.1	3.7	60.5	11.4	0.0138$^{*}$
12.	MAN	135	6	0.8	78.9	11.4	163	5	2.5	23.7	$5.4^{*}$	164	3	3.3	42.1	$8.6^{*}$	3.3	36.8	$7.9^{*}$	0.0000$^{*}$
13.	MUV	136	6	5.0	44.7	10.4	115	6	3.3	34.2	$7.5^{*}$	126	2	1.7	42.1	$7.1^{*}$	2.9	42.1	$8.2^{*}$	0.0000$^{*}$
14.	NED	136	6	2.5	73.7	12.1	106	5	3.3	60.5	11.1	147	3	1.7	84.2	12.9	5.8	71.1	14.6	0.0009$^{*}$
15.	OTS	129	6	0.8	94.7	13.6	131	3	0.8	89.5	12.9	136	2	0.8	100.0	14.3	0.8	97.4	13.9	0.6049
16.	RER	129	6	1.2	60.5	9.3	139	4	1.2	31.6	$5.4^{*}$	146	4	0.8	63.2	9.3	2.9	36.8	$7.5^{*}$	0.0000$^{*}$
17.	ROC	135	6	5.8	47.4	11.4	124	4	1.2	31.6	$5.4^{*}$	136	3	0.0	68.4	9.3	0.8	36.8	$5.7^{*}$	0.0000$^{*}$
18.	ROM	132	6	1.7	73.7	11.4	127	5	2.1	44.7	$7.9^{*}$	119	4	4.1	52.6	10.7	2.5	50.0	$8.9^{*}$	0.0000$^{*}$
19.	SCA	137	6	3.3	86.8	14.6	120	5	1.7	71.1	11.1	119	5	12.4	84.2	22.1	2.9	86.8	14.3	0.0633
20.	SCH	131	6	2.5	71.1	11.8	127	7	2.1	34.2	$$6.4^{*}$$	142	5	0.8	68.4	10.0	2.5	50.0	$$ 8.9^{*}$$	0.0000$$^{*}$$
21.	SCK	142	6	0.8	63.2	$9.3^{*}$	151	8	1.2	18.4	$3.6^{*}$	182	5	0.8	42.1	$6.4^{*}$	3.3	31.6	$7.1^{*}$	0.0000$^{*}$
22.	SCT	132	6	1.2	68.4	10.4	149	8	0.8	47.4	$$7.1^{*}$$	140	6	2.5	68.4	11.4	0.8	47.4	$$ 7.1^{*}$$	0.0001$$^{*}$$
23.	SCW	135	6	3.7	84.2	14.6	156	12	2.9	34.2	$7.1^{*}$	163	4	4.1	89.5	15.7	5.8	52.6	12.1	0.5310
24.	UMD	124	6	1.2	57.9	$$8.9^{*}$$	138	5	0.8	28.9	$$4.6^{*}$$	135	4	0.8	36.8	$$ 5.7^{*}$$	1.7	34.2	$$6.1^{*}$$	0.0000$$^{*}$$
25.	USD	137	6	8.3	57.9	15.0	139	7	2.5	50.0	$8.9^{*}$	156	3	2.5	73.7	12.1	3.3	63.2	11.4	0.0012$^{*}$
26.	WIB	125	6	0.8	73.7	10.7	127	7	1.7	28.9	$$5.4^{*}$$	133	5	0.0	42.1	$$ 5.7^{*}$$	2.5	42.1	$$7.9^{*}$$	0.0000$$^{*}$$
27.	ZIA	124	6	0.4	92.1	12.9	147	10	2.1	34.2	$6.4^{*}$	159	5	8.3	78.9	17.9	2.5	57.9	$10.0^{*}$	0.0748
Group averages:				2.6	74.7	12.4			2.2	45.4	8.0			3.1	64.3	11.4	3.0	55.0	10.0	
* Significant at level $\varvec{\alpha }$ = 0.05:						4					18					8			14	19

Hotelling’s T$^{2}$-tests were performed with only 9 deviant and 50 standard trials (of each dataset)

All t-CWT computations were performed with the t-CWT software [16, 17] in 64-bit MATLAB 8.1.0.604 (R2013a), under GNU Linux (Scientific Linux 6), on the high performance computing cluster bwGRiD [31] using a single quad-core CPU (Intel${}^{\circledR }$ Xeon${}^{\circledR }$ 5150 @ 2.66 GHz) per job (i.e. for a particular combination of input parameters). The t-CWT program was also tested with 64-bit MATLAB 8.3.0.532 (R2014a) and 64-bit GNU Octave 3.8.2 [15], under GNU Linux (openSUSE 12.3) and Windows 7, on less powerful desktop and laptop computers (equipped with dual-core CPUs).

## Results and discussion

In this section, some example results are presented and discussed. These results were obtained with the t-CWT method from the example datasets [18] described above.

### Results from the example oddball data

Group averages of individual hold-out classification error rates for different values of the cutoff scale $S_c$, the log-grid sampling rate *R*, and the percentage of variance $P\!_v$ explained by PCA are displayed in Table 2. Most of these results are visualized by bar plots in Fig. 3. Both total errors (tot) and errors for each category of trials, standard (std) and deviant (dev), are displayed. The total error rates computed by taking into account the *a priori* oddball probabilities $p_s$ = 86.4 % and $p_d$ = 13.6 % (15) were, as expected, much smaller than those computed for $p_s\!=\!p_d$ =50 % (14). Note, however, that the error rates for deviant trials increased when the *a priori* probabilities were taken into account. It is also interesting to point out the following observation: while for $p_s$ = 86.4 % and $p_d$ = 13.6 %, the total error (Fig. 3f) was decreased by the error reduction in the classification of deviant trials (Fig. 3e), the corresponding total decrease in the case of equal *a priori* probabilities $p_s\!=\!p_d$ =50 % (Fig. 3c), was caused by the decline of error of classification of standard trials (Fig. 3a).

Both the numbers in Table 2 and the plots in Fig. 3 demonstrate how the quality of the LDF $\mathbf {d^\star }$ (52–56) can be optimized by minimizing the errors of classification through systematic variation of the values of the different input parameters $S_c$, $P\!_v$, *R*, etc.. This optimization can be very important for different applications of the t-CWT method. While this is obvious for cases in which classification is the ultimate goal of the application (as, e.g., in BCI), other applications aimed at multivariate hypothesis testing may also use optimized LDFs for their purposes (see the “Discussion” section).

In Table 2, $N\!_F$ denotes the number of frequency components per channel (31, 32), and $N\!_G$ is the number of log-grid vertices (42). The number of non-zero CWT matrix elements (ME) is given by $K N\!_F N\!_G$, where *K* is the number of channels. Table 2 shows that the ME ratio $(N\!_F N\!_G S_c^2)/(R^2 T^2)$ (see 46) is approximately constant. Table 2 also shows how the computational time depends on the number of non-zero CWT matrix elements $K N\!_F N\!_G$ which is a function of $S_c$, *R*, $P\!_v$, and the number of channels *K*. This function is defined by (59) which was confirmed by the results displayed in Table 2. The computational demands as functions of $S_c$, $f_c$ and *R* are presented in graphical form in Fig. 3g-i.

The total processing time for the hold-out method is the product of the processing time per non-zero CWT element, the number of such elements, and the number of scalograms. For the hold-out method, as applied to the example data, the latter was equal to the total number of trials = 27 datasets $\times$ 280 trials per dataset = 7,560. As the last column of Table 2 shows, the processing time per matrix element is approximately constant. Note, however, that this value of less than a half microsecond was obtained with MATLAB and certain hardware (see above), and that the corresponding value for GNU Octave and/or other hardware may be larger.

The results for $S_c$ = 40 ms, *R* = 15 pps, and $P\!_v$ = 99 % are presented in more detail. The latter values appeared to be optimal, because, as Table 2 and Fig. 3i show, larger values of *R* increased substantially the computational demands with little improvement of the results.

The average ERP curves are displayed in Fig. 2a for the whole group (grand average) and in Fig. 2b for one individual participant (ID=‘GIM’). Student’s t-test results for the difference between the ERP responses to deviant vs. standard tones are displayed in Fig. 2c for the whole group and in Fig. 2d for the participant ‘GIM’. Note that, since these t-values were not corrected for multiple comparisons, they cannot be used for inference about the statistical significance of the difference between the two ERP curves at each point in time [4, 5]. It is interesting, however, to compare the *forms* of these t-value curves with the forms of the corresponding LDFs. The LDF obtained with the t-CWT method is displayed in Fig. 2e for the whole group and in Fig. 2f for the participant ‘GIM’.

The grand average plots show that the ERP response to deviant stimuli relative to the response to standards was dominated by the Mismatch Negativity (peaking about 200 ms post-stimulus) [32], the P3 (300 ms) [19, 20], and the Negative Slow Wave (400 ms) [33]. The individual LDF displayed in Fig. 2f shows, however, some additional oscillatory features which are not quite discernible in the time domain (Fig. 2b, d) and which, nevertheless, improve substantially the LDA classification results obtained with this LDF for the participant ‘GIM’ compared to those computed for the same participant with the group LDF displayed in Fig. 2e (see Table 3).

The different LDA error rates as well as the *p*-values obtained from Hotelling’s T${}^2$-test (with 9 deviant and 50 standard trials) for each participant, for $S_c$ = 40 ms, *R* = 15 pps, and $P\!_v$ = 99 % are displayed in Table 3. Total error rates (tot) were tested by the binomial cumulative distribution function whether they were significantly smaller than the *a priori* probability for deviant (dev) trials $p_d=$ 13.6 %. The comparison between the different methods shows an advantage of the classifications based on individual t-CWT features over those based on group features. Furthermore, the individual hold-out method provided lower error rates than the split-half method. This can be explained by the split-half method’s loss of statistical power (less that 20 deviant trials remained after splitting a dataset).

Table 3 also shows the number of t-CWT extrema $N\!_X$ obtained with each method for each data set and the number of SPCs used for classification. While the error rates obtained with the individual biased method are incorrect, the numbers of t-CWT features $N\!_X$ and the numbers of SPCs $N\!_P$ are the correct numbers obtained from the whole datasets. They can also be seen as good approximations for the corresponding values obtained with the holdout-method, in which, of course, they vary with each iteration (and are not displayed in Table 3 for this reason). These numbers show that the originally very large dimensionality of the data was reduced to less than 200 (correlated) t-CWT extrema which in turn were reduced further to a few (12 or less, uncorrelated) SPCs. The results from Hotelling’s T${}^2$-test (Table 3, last column) show that, for the most individual datasets, 9 deviants and 50 standards were enough for the ERP difference to reach statistical significance.

### Discussion

Table 3 shows that all error rates vary largely among the different individual datasets. While the inherent EEG noise in the data is responsible for one portion of the error, participants’ inattention to the stimuli provides an additional source of systematic error. Thus, error rates can be used as inverse measures of attention.

A much more straightforward approach, however, would be to use directly the LDF value $\mathbf {vd^\star }$ (52–57) to measure the ERP difference between the two experimental conditions and to interpret this difference as a measure of participants’ attention to the stimuli. This could be done in different ways as described in the following three cases.

In the first case, both $\mathbf {v}$ and $\mathbf {d^\star }$ are obtained from the same ERP sample $\mathbf {V}$, comprising two subsamples $\mathbf {V}_{\!\!_\mathcal {A}}$ and $\mathbf {V}_{\!_\mathcal {B}}$ obtained under two different experimental conditions $\mathcal {A}$ and $\mathcal {B}$ ($\mathcal {A}$-$\mathcal {B}$ design). Then, the mean difference LDF value $\left( \mathbf {\overline{v}}_{\!\!_\mathcal {A}}\!-\mathbf {\overline{v}}_{\!_\mathcal {B}}\right) \mathbf {d^\star }$ is exactly the mean Mahalanobis distance (10–12) computed in the feature space. This same Mahalanobis distance is used in Hotelling’s T${}^2$-test, which, however, should not be used directly in this case, because the t-CWT features are extracted from the very sample that is tested. As already mentioned in the Background section, a multivariate randomization test based on Hotelling’s T${}^2$-statistic can be used instead [11].

In the second case, the LDF $\mathbf {d^\star }$ is computed from the same sample as $\mathbf {v}$, but, this time, using t-CWT features obtained from a training dataset, $\mathbf {V}_{\!\!_\mathcal {Z}}$ (by an $\mathcal {A}$-$\mathcal {B}$ design within $\mathcal {Z}$). In this case, Hotelling’s T${}^2$-test may be used, as this was done above in the assessment of the example data.

The third case, in which both the t-CWT features and the LDF $\mathbf {d^\star {\!}_{\!\!_\mathcal {Z}}}$ are computed from a training dataset $\mathbf {V}_{\!\!_\mathcal {Z}}$, while $\mathbf {v}$ is drawn from a test dataset $\mathbf {V}$, is the most important and will be discussed in more detail. As already mentioned above, this is the usual scenario in a typical BCI application, which was also demonstrated above by the split-half method in the assessment of classification error rates. But the representation of the Mahalanobis distance (12) suggests another usage of the LDF apart from single trial classification. Namely, we can construct a new estimator $D^2_{\!\!_\mathcal {Z}}$ of the Mahalanobis distance by using $\mathbf {d^\star {\!}_{\!\!_\mathcal {Z}}}$ instead of $\mathbf {d}$:

60 $$\begin{aligned} D^2_{\!\!_\mathcal {Z}}( \mathbf {v}_{\!\!_\mathcal {A}}, \mathbf {v}_{\!_\mathcal {B}}) = \left( \mathbf {v}_{\!\!_\mathcal {A}}- \mathbf {v}_{\!_\mathcal {B}}\right) \,\mathbf {d^\star {\!}_{\!\!_\mathcal {Z}}}. \end{aligned}$$

Since $\mathbf {d^\star {\!}_{\!\!_\mathcal {Z}}}$ is computed from the training dataset $\mathbf {V}_{\!\!_\mathcal {Z}}$, it can be treated as constant in all tests and analyses concerning the test dataset $\mathbf {V}$. Consequently, $\mathbf {v}_{\!\!_\mathcal {A}}\mathbf {d^\star {\!}_{\!\!_\mathcal {Z}}}$, $\mathbf {v}_{\!_\mathcal {B}}\mathbf {d^\star {\!}_{\!\!_\mathcal {Z}}}$, and $D^2_{\!\!_\mathcal {Z}}(\mathbf {v}_{\!\!_\mathcal {A}},\mathbf {v}_{\!_\mathcal {B}})$ are all (univariate) normally distributed and can be subjected to standard univariate tests. It should also be noted that in (60) a whole complex pattern recognition scheme derived from $\mathbf {V}_{\!\!_\mathcal {Z}}$ (PCA, t-CWT, etc.) is imposed on $\mathbf {V}$ by simple multiplication.

The significance of (60) reaches, however, beyond mere computational convenience, because it conveys a whole concept of multivariate ERP assessment. While, in most BCI applications, this concept is clearly the most effective for single trial classification, and, therefore, also the standard one, it has not been used in other ERP applications for testing hypotheses about mean ERP differences. (Note that mass univariate analyses [4, 5], which have become popular recently, represent a different concept.) In the following, some general ideas about possible applications of this multivariate concept are presented.

The two kinds of t-CWT features, group and individual, that can be extracted from the data suggest two different kinds of diagnostic applications of the t-CWT method: within-subject and between-subject. Consider an ERP paradigm testing a certain cognitive function in a group of individuals under three different conditions: $\mathcal {X}$, experimental condition (e.g. under the influence of a drug); $\mathcal {Y}$, control condition (e.g. placebo); and $\mathcal {Z}$, standard condition (no substances administered). Assume that the cognitive function of interest is reflected by the ERP difference between two sub-conditions, $\mathcal {A}$ and $\mathcal {B}$, ($\mathcal {A}$-$\mathcal {B}$ design, as above). Now, the *individual* t-CWT features, SPCs, and LDFs obtained from $\mathcal {Z}$ can be used to assess the difference between $\mathcal {X}$ and $\mathcal {Y}$ by means of Student’s two-sample ($\mathcal {X}$-$\mathcal {Y}$) t-test of the Mahalanobis distance (60) and/or by comparison of classification error rates. This is an example of a within-subject application of the t-CWT method. Note that the t-CWT method described above for the case of an $\mathcal {A}$-$\mathcal {B}$ design can be easily extended to the case of only one sub-condition, $\mathcal {A}$, when the ERP $\mathbf {v}$ is compared to **0** ($\mathcal {A}$-0 design).

As an example of a between-subject application, consider an ERP paradigm testing a certain cognitive function in three different samples of individuals: $\mathcal {X}$, a sample of patients suffering a certain disorder which has the impairment of this cognitive function as a symptom; $\mathcal {Y}$, a sample of healthy individuals; and $\mathcal {Z}$, a mixed sample of patients and healthy individuals. In this case, the *group* t-CWT features, SPCs, and LDFs obtained from $\mathcal {Z}$ can be used to assess the difference between $\mathcal {X}$ and $\mathcal {Y}$. Further, the t-CWT method can be applied directly to the ERP difference between $\mathcal {X}$ and $\mathcal {Y}$ and the resulting t-CWT features, SPCs, and LDFs can be used for classification and diagnostics of individuals from the general population (i.e. to determine whether an individual suffers from this particular symptom or not). The greatest problem in the case of a between-subject comparison would be the substantial increase in variance due to individual differences.

Finally, in both within-subject and between-subject applications, the LDF value (54–57) can be used as a measure of the magnitude of the (differential) ERP response in each of the conditions $\mathcal {X}$, $\mathcal {Y}$, and $\mathcal {Z}$ in order to investigate its relationship with other behavioral measures or with the amplitudes of single (classical) ERP components.

#### Limitations

All of the above ideas about possible applications of the t-CWT method assume the existence of a cleverly designed ERP paradigm that tests *exclusively* (changes in) a particular (set of) cognitive function(s) of interest without the resulting ERP differences being affected by (changes in) other cognitive functions. In many cases, however, this assumption might not be true. This is a conceptual limitation, not only of the t-CWT method, but of any multivariate ERP assessment method. In certain cases, the amplitudes of single ERP components might be better measures of particular cognitive functions of interest.

A purely technical limitation of the t-CWT method is imposed by the computational demands of its current software implementation [16, 17]. For instance, the usage of dense electrode arrays combined with long time windows and small cutoff scales, might result in practical unusability of the application, if no access to adequate computational resources (e.g. a high performance computing cluster [31]) is provided. In spite of the good scalability of the application (e.g. the computationally demanding CWT is performed one channel at a time, the maximum number of single trials processed by CWT is controlled by an input parameter, thus limiting working memory usage), the increase of computational time can be handled only by parallel execution of t-CWT jobs on several powerful CPUs.

A notable limitation of the current study is the lack of evidence for (or against) the usefulness of the novel PCA-based multivariate outlier detection procedure introduced above. The results of the (within-subject) assessment of the example oddball data were not sensitive to variations in the number of outliers detected and excluded by the procedure, depending on the value of the input parameter *C* (35). Whether zero or as much as 20 % of the trials were rejected, the statistical assessments were practically not affected by these changes and the corresponding results remained virtually the same. It should be mentioned, however, that some unpublished evidence already exists, suggesting that the outlier detection procedure could be crucial in a between-subject design. This evidence comes from the (unfinished) t-CWT assessment of ERP data obtained in a previous study [34].

## Conclusions

In the present article, some basic concepts of multivariate statistics were introduced as geometric notions. ERPs were defined as random vectors (points) in a metric space, in which the distance between two points was derived in a natural way from the covariance of the data. PCT and DFT were introduced as rotations in this space. LDA classification was described as computing a LDF vector, building a separation plane perpendicular to this vector, and assigning single-trial ERP points to two different categories according to their position relative to this dividing plane. CWT and t-CWT were also defined as linear transformations represented by their respective matrices. All these mathematical constructs were used to provide for the first time a detailed, step-by-step, formal description of the t-CWT algorithm. Its MATLAB and GNU Octave implementation was also made publicly available as free and open source code released for the first time under GPLv3 [16, 17].

A new multivariate outlier rejection procedure based on PCA in the frequency domain was introduced. The time-dependent filtering and the DWT used in the previous version of t-CWT [7] were replaced in the current version by simple uniform filtering via DFT. This was done solely for simplification for the sake of easier understanding and *not* for improvement of the method. In fact, a new version of t-CWT is planned including a more flexible procedure for time-dependent filtering based on PCA and DWT.

It should be noted that t-CWT is essentially a feature extraction method and t-CWT based classification does not necessarily imply LDA as a post-processing procedure. Classification can be performed using other methods as well, e.g., SVM [10]. Hypotheses can be tested by both mass-univariate [13] and multivariate [11] permutation/randomization tests. Moreover, as already mentioned above, t-CWT can also be used in combination with other dimensionality reduction methods, e.g. EMS filtering [24]. On the other hand, the PCA-based multivariate outlier detection introduced here, can be used independently from t-CWT as a pre-processing procedure in other assessment algorithms. It is also important to emphasize that although t-CWT feature extraction can be computationally demanding, taking several seconds or even minutes for large scalograms (with many channels), the t-CWT features, once computed, can be applied practically instantly in real time applications (e.g., BCI) via LDA or other proper classification method.

The t-CWT method was demonstrated on example ERP data [18] obtained in a passive oddball paradigm. Both group and individual t-CWT features were extracted from the data and were used for LDA classification of single trials and for testing mean ERP differences for each individual dataset via Hotelling’s T${}^2$-test. Different methods for estimation of classification errors were introduced and compared with each other.

Finally, new ideas for further applications of the multivariate approach in general and of t-CWT method in particular were introduced on a conceptual level in the Discussion. Some of these ideas will be tested soon in a randomized clinical trial where ERPs are used for assessment of the sustained mindful attention developed by training in a course of mindfulness-based cognitive therapy for recurrent depression [34].

## Availability of supporting data

The example datasets [18] supporting the results of this article are available at http://tcwt.de/ or http://bioinformatics.org/tcwt/ as well as in the LabArchives repository at http://doi.org/10.6070/H4MP518T.

## Additional file

10.1186/s12868-015-0185-z **t-CWT 2.01: A software implementation of the t-CWT method for multivariate assessment of event-related potentials.** A ZIP archive (t-CWT.2.01.zip) of the t-CWT free and open source code for MATLAB and GNU Octave [16] released under GPLv3.

## Abbreviations

BCI: brain–computer interface
CPU: central processing unit
CWT: continuous wavelet transform
DFT: discrete fourier transform
DWT: discrete wavelet transform
EEG: electroencephalogram
EMS: effect-matched spatial (filtering)
ERP: event-related potential
GPLv3: GNU general public license, version 3
LDA: linear discriminant analysis
LDF: linear discriminant function
PCA: principal component analysis
PCT: principal component transform
SCP: selected principal component (by step down selection)
SS: sums of squares (for computation of sample variance)
SVM: support vector machines
