# Supplementary material for: Multivariate assessment of event-related potentials with the t-CWT method
Source: BMC Neurosci. 2015 Nov 5;16:73. doi: 10.1186/s12868-015-0185-z (PMC4635610; doi:10.1186/s12868-015-0185-z)
Supplement: Supplementary file 1 — 10.1186/s12868-015-0185-z t-CWT 2.01: A software implementation of the t-CWT method for multivariate assessment of event-related potentials. A ZIP archive (t-CWT.2.01.zip) of the t-CWT free and open source code for MATLAB and GNU Octave [16] released under GPLv3. [file 12868_2015_185_MOESM1_ESM.zip › t-CWT.2.01/t-CWT.2.01.help.html]

t-CWT 2.01 Documentation


# t-CWT 2.01 Documentation

```
Copyright © 2003-2015 Vladimir Bostanov <tcwt_help use>

This HTML file was automatically generated from the
t-CWT help texts contained in the source code files.

t-CWT is a method for multivariate statistical assessment of event-related
brain potentials (ERP). The help texts describe the t-CWT software. The
underlying mathematical algorithm is described elsewhere <tcwt_help math>.
```

## Table of Contents

- help
- use
- math
- install
- intro
- example
- prm
- var
- scr
- prm2mat
- prm2info
- dir2dsnl
- dsnl2xdsnl
- ascii2tmat
- t2f
- f2merge
- f2split
- f2pool
- f2pc
- f2cwss
- ri2ri1out
- pc2cnd2ri
- f2x
- x2ld
- f2stats
- f2holdout
- f2plot
- ld2plot
- gpl

## Themes

### help

```
 tcwt_help        invoked with no arguments displays this text;
 tcwt_help main   also displays this text;
 tcwt_help help   does the same.
 tcwt_help NAME   displays help text for the script or function tcwt_NAME.

 t-CWT version 2.01

 Copyright (C) 2003-2015 Vladimir Bostanov <http://tcwt.de/>

 t-CWT comes with ABSOLUTELY NO WARRANTY. This is free software, and you
 are welcome to redistribute it under certain conditions. See the file
 tcwt_use.m <tcwt_help use>. For more details, see the file COPYING or
 tcwt_gpl.m <tcwt_help gpl>. If not, see <http://www.gnu.org/licenses/>.

 t-CWT is a method for multivariate statistical assessment of event-related
 brain potentials (ERP). The help texts describe the t-CWT software. The
 underlying mathematical algorithm is described elsewhere <tcwt_help math>.

 DOWNLOAD, INSTALLATION AND CONFIGURATION: <tcwt_help install>

 GETTING STARTED: <tcwt_help intro>

Table of Contents
```

### use

```
 TERMS OF USE

 t-CWT Copyright (C) 2003-2015 Vladimir Bostanov <http://tcwt.de/>

 t-CWT is free software: you can redistribute it and/or modify
 it under the terms of the GNU General Public License as published
 by the Free Software Foundation, either version 3 of the License,
 or (at your option) any later version.

 t-CWT is distributed in the hope that it will be useful,
 but WITHOUT ANY WARRANTY; without even the implied warranty
 of MERCHANTABILITY or FITNESS FOR A PARTICULAR PURPOSE.
 See the GNU General Public License for more details.

 You should have received a copy of the GNU General Public License
 along with t-CWT; see the file COPYING or the file tcwt_gpl.m
 <tcwt_help gpl>. If not, see <http://www.gnu.org/licenses/>.


Table of Contents
```

### math

```
 t-CWT MATH

 A detailed description of the mathematical algorithm implemented
 by the t-CWT software and the results from the assessment of the
 example ERP data <tcwt_help example> can be found in

    Bostanov V. (2015). Multivariate Assessment of Event-Related
    Potentials with the t-CWT Method. BMC Neuroscience.
    DOI: 10.1186/s12868-015-0185-z
    <http://doi.org/10.1186/s12868-015-0185-z>.

 The first version of t-CWT was originally introduced in

    Bostanov V. (2004). BCI Competition 2003 -- data sets Ib and IIb:
    feature extraction from event-related brain potentials with
    the continuous wavelet transform and the t-value scalogram.
    IEEE Trans Biomed Eng 51:1057-61.

 If you use t-CWT in your research, please cite Bostanov (2015),
 or both Bostanov (2015) and Bostanov (2004).

 BibTeX ENTRIES:

    @article{bostanov15tcwt,
       author={Bostanov, Vladimir},
       title={Multivariate Assessment of Event-Related Potentials
          with the {t-CWT} Method},
       journal={BMC Neuroscience},
       note={DOI: 10.1186/s12868-015-0185-z},
       year={2015}}

    @article{bostanov04tcwt,
       author={Bostanov, Vladimir},
       title={{BCI} {C}ompetition 2003 - Data Sets {I}b and {II}b:
          {F}eature Extraction from Event-Related Brain Potentials with
          the Continuous Wavelet Transform and the t-Value Scalogram},
       journal={IEEE Trans Biomed Eng},
       volume={51},
       pages={1057-1061},
       year={2004}}

Table of Contents
```

### install

```
 DOWNLOAD, INSTALLATION AND CONFIGURATION: May the Source be with you!

 1. Download the t-CWT source code from <http://tcwt.de/>
    or <http://bioinformatics.org/tcwt/>.
    You can also download the example ERP data from <http://tcwt.de/>, or
    <http://bioinformatics.org/tcwt/>, or <http://doi.org/10.6070/H4MP518T>.

 2. Unpack the t-CWT source code archive file at the desired location
    in your file system.

 3. Open tcwt_setup.m and change the value of tcwtROOT to the full path
    of the t-CWT root directory according to the installation in step 1.

 4. Set the value of tcwtWORK in tcwt_setup.m. tcwtWORK is the full path
    to the location in your file system where your input and output ERP
    data will be stored.

 5. Start MATLAB or GNU Octave in interactive mode, change directory to
    the t-CWT root directory, and type "tcwt_setup". You should get the
    t-CWT Copyright message confirming that t-CWT is ready for use.

 6. Add the statements in tcwt_setup.m to your MATLAB startup.m and/or
    to your GNU Octave octaverc file, if you wish to have t-CWT ready
    for use at startup (without having to type "tcwt_setup" every time).

    NOTE: If t-CWT jobs are executed in MATLAB, some t-CWT functions
          use probability distributions from the Statistics Toolbox.

Table of Contents
```

### intro

```
 INTRODUCTION TO t-CWT: The Eightfold Path

 1. Read this introduction, then download and assess the example ERP data
    <tcwt_help example> to get a first impression of how t-CWT works.
    Then, create your own project following the steps below.

 2. Choose a new project name (prjNAME) and make a directory
    tcwtWORK/prjNAME (Here and further, tcwtROOT and tcwtWORK denote
    the t-CWT root path and the t-CWT work path <tcwt_help install>.)

 3. Perform EOG correction, technical artifact rejection, segmentation and
    binary-to-ASCII conversion of your ERP data with some other software,
    and save the obtained ASCII datasets to tcwtWORK/prjNAME/_ascii.

 4. Choose a new job input parameter file name (prmNAME) and copy
    the demo job input parameter file tcwtROOT/demo/tcwt_prm.m to
    tcwtROOT/jobs/prjNAME_prmNAME.m <tcwt_help prm>. Then, open
    prjNAME_prmNAME.m with an appropriate text editor and make the
    necessary changes. For instance, set ASCIIext, isMultiplex, the
    analog-to-digital time-sampling rate a2dR, etc. according to the
    results from the binary-to-ASCII conversion <tcwt_help ascii2tmat>.

 5. Choose a new job input variable file name (varNAME) and copy
    the demo job input variable file tcwtROOT/demo/tcwt_var.m to
    tcwtROOT/jobs/prjNAME_varNAME.m <tcwt_help var>. Then, open
    prjNAME_varNAME.m and make the necessary changes. Change, e.g.,
    the PCA criteria, the outlier criteria, etc. <tcwt_help f2pc>.

 6. Choose a new job script file name (scrNAME) and copy
    the demo job script file tcwtROOT/demo/tcwt_scr.m to
    tcwtROOT/jobs/prjNAME_scrNAME.m <tcwt_help scr>. Then,
    open prjNAME_scrNAME.m and make changes, if necessary.

 7. Copy the demo main script file tcwtROOT/demo/tcwt_scr_prm_var.m to
    tcwtROOT/jobs/prjNAME_scrNAME_prmNAME_varNAME.m and then just run
    prjNAME_scrNAME_prmNAME_varNAME with MATLAB or with GNU Octave, either
    in interactive mode, or from the command line, or from a shell script,
    e.g.: matlab -r "myprj_scr_prm_var" -logfile myprj_scr_prm_var.log
    or:   octave --eval "myprj_scr_prm_var" &> myprj_scr_prm_var.log
    The main script extracts scrNAME, prmNAME, and varNAME from its own
    name, calls the corresponding scripts and saves the results of their
    execution in the directory tcwtWORK/prjNAME/prmNAME_varNAME (which is
    newly created by the main script, if it does not exist).

 8. In order to view the results from the t-CWT assessment of your ERP
    data, open the file tcwtWORK/prjNAME/prmNAME_varNAME/_A_run_scrNAME.log

Table of Contents
```

### example

```
 t-CWT EXAMPLE: Oddball

 1. Download the example oddball ERP data <tcwt_help install>.

 2. Unpack the archive file in tcwtWORK/example/_ascii. (Here and
    further, tcwtROOT and tcwtWORK denote the t-CWT root path and
    the t-CWT work path, respectively <tcwt_help install>.)

 3. Read the Introduction to t-CWT <tcwt_help intro>.

 4. Run the job example_full_s250r10_pca95 to check whether t-CWT is
    working fine. This should take 10 minutes or less with MATLAB
    and a little longer if you are using GNU Octave.

 5. Run the script example_full with other values of the input parameters
    <tcwt_help prm> and the input variables <tcwt_help var>, starting
    with larger values of cutoffScale and smaller values of logGridR and
    progressing to more computationally demanding settings. You will find
    ready main scripts for several combinations of values in tcwtROOT/jobs.

 6. Use the output of the function tcwt_prm2info <tcwt_help prm2info> and
    the "Elapsed time" value from the log file of a previously executed job
    (e.g., tcwtWORK/example/s100r15_pca95/_A_run_full.log) before running
    a new job, if you want to estimate the execution time of the new job.

 7. Compare the results you got to those published in the journal article
    reporting on the t-CWT assessment of the example data <tcwt_help math>.

Table of Contents
```

### prm

```
 A t-CWT job input parameter file prjNAME_prmNAME is called from
 the main job script prjNAME_scrNAME_prmNAME_varNAME <tcwt_help main>.
 The job input parameters are job constants <tcwt_help prm2mat>.

 The main script saves a copy of the job input parameter
 file tcwtROOT/jobs/prjNAME_prmNAME.m in './_A_prm_prmNAME.m'
 (i.e. in tcwtWORK/prjNAME/prmNAME_varNAME/_A_prm_prmNAME.m).

 Create a new input parameter file prjNAME_prmNAME by copying
 the demo input parameter file tcwtROOT/demo/tcwt_prm.m
 to tcwtROOT/jobs/prjNAME_prmNAME.m and editing the copy.

 Use tcwt_prm2info(prjNAME_prmNAME) <tcwt_help prm2info>
 to estimate the approximate computational demand of the job
 that should be executed with these input parameter settings.

 JOB INPUT PARAMETERS

 Window = [time1 time2];
 Beginning and end (in seconds) of the time window
 in which ERP assessment should be performed.
 Window must be within the range defined by Epoch (see below).

 cutoffScale = time;
 Cutoff scale in seconds.

 logGridR = number;
 Log-grid sampling rate in points per scale (pps).

 BaseLine = [time1 time2];
 Beginning and end (in seconds) of the baseline job constants
 to which the EEG in Window will be referenced.

 fadeInOut = [time1 time2];
 Fadein and Fadeout time for the Tukey window function.

 NcwMax = number;
 Maximum number ot trials processed by CWT in one step.
 If there are many trials to be processed and NcwMax is too big
 t-CWT may run out of memory.

 Epoch = [time1 time2];
 Beginning and end (in seconds) of EEG epoch, defined by the input data.

 NtimePt = number;
 Number of time sampling points in the Epoch.
 For an ERP ASCII file 'dataset.dat' exported from Brain Broducts EEG
 format, the value of NtimePt can be obtained from 'dataset.vhdr'.
 If NtimePt <= 0, its value is ignored. If both a2dR > 0 and NtimePt > 0,
 their values must be in the right relationship with each other and
 with the length of the EEG epoch.

 a2dR = number;
 Analog-to-digital time-sampling rate in Hz, defined by the input data.
 For an ASCII ERP file 'dataset.dat' exported from Brain Broducts EEG
 format, the value of a2dR can be obtained from 'dataset.vhdr'.
 If a2dR <= 0, its value is ignored. If both a2dR > 0 and NtimePt > 0,
 their values must be in the right relationship with each other and
 with the length of the EEG epoch.

 ASCIIext = string;
 File extension (without the leading '.')
 of the ASCII ERP datasets that will be imported into t-CWT.

 isMultiplex = 0 or 1;
 isMultiplex tells t-CWT whether the imported ASCII data are in vectorized
 (isMultiplex = 0) or in multiplexed format <tcwt_help ascii2tmat>. For an
 ASCII ERP file 'dataset.dat' exported from Brain Broducts EEG format,
 this information can be obtained from 'dataset.vhdr'.

 ChannelList = ['ChannelName1 '; 'ChannelName2 ';... 'ChannelNameN~';... ];
 A character array whose rows are the names of the EEG channels. Both
 the names and the order of the channels are defined by the input data.
 For an ERP ASCII file 'dataset.dat' exported from Brain Broducts EEG
 format, the channels can be obtained from 'dataset.vhdr'. Only channels
 whose names end with a blank are processed; the rest is discarded.
 Change channel names to tell t-CWT which channels should be processed,
 but don't change the order defined by the imported ERP data!

Table of Contents
```

### var

```
 A t-CWT job input variable script prjNAME_varNAME is called from
 the main job script prjNAME_scrNAME_prmNAME_varNAME <tcwt_help main>.

 Job input variables define all input factors which are not defined by
 input parameters <tcwt_help prm>, e.g., PCA criteria, outlier criteria,
 etc. Unlike job input parameters, input variables are passed to a t-CWT
 function directly through the function arguments when the function
 is called from the job script prjNAME_scrNAME <tcwt_help scr>.

 The main script saves a copy of the job input variable
 file tcwtROOT/jobs/prjNAME_varNAME.m in './_A_var_varNAME.m'
 (i.e. in tcwtWORK/prjNAME/prmNAME_varNAME/_A_var_varNAME.m).

 Create a new input variable file prjNAME_varNAME by copying
 the demo input variable file tcwtROOT/demo/tcwt_var.m
 to tcwtROOT/jobs/prjNAME_varNAME.m and editing the copy.

Table of Contents
```

### scr

```
 A t-CWT job script prjNAME_scrNAME is called from the main
 job script prjNAME_scrNAME_prmNAME_varNAME <tcwt_help main>.
 The job script is executed in the project directory
 tcwtWORK/prjNAME/prmNAME_varNAME, where tcwtWORK
 denotes the t-CWT work path <tcwt_help install>

 A job script is mostly a list of function calls to t-CWT functions, whose
 source files are in tcwtROOT/func/ (tcwtROOT denotes the t-CWT root path).

 t-CWT functions using one or more job constants access them by loading
 the file './_A_const.mat' <tcwt_help prm2mat>. Job constants are computed
 and stored in './_A_const.mat' by the main script before calling the
 job script. They are computed from the job input parameters defined
 by the input parameter script prjNAME_prmNAME <tcwt_help prm>.

 The main script calls also the input variable script prjNAME_varNAME.
 Unlike job input parameters, job input variables are passed to t-CWT
 functions directly through the function arguments <tcwt_help var>.

 The main script saves a copy of the job script file
 tcwtROOT/jobs/prjNAME_scrNAME.m in './_A_scr_scrNAME.m'
 (i.e. in tcwtWORK/prjNAME/prmNAME_varNAME/_A_scr_scrNAME.m).

 Create a new job script prjNAME_scrNAME by simply copying
 the demo job script file tcwtROOT/demo/tcwt_scr.m to
 tcwtROOT/jobs/prjNAME_scrNAME.m and editing the copy.

Table of Contents
```

### prm2mat

```
 tcwt_prm2mat(prmFILE) reads job input parameters <tcwt_help prm> from
 the file prmFILE.m, computes other job constants and saves all of them
 into the file './_A_const.mat'. The term 'job constants' means that,
 once defined, these variables do not change any more during job execution.
 This definition includes all job input parameters and all variables
 computed from them by tcwt_prm2mat which is called from the main script
 <tcwt_help main> before calling the job script <tcwt_help scr>.
 During job execution, t-CWT functions using one or more job
 constants access them by loading the file './_A_const.mat'.

 JOB CONSTANTS

   fullTime   A vector consisting of the time-sampling points defined
              by the job input parameters a2dR and Epoch <tcwt_help prm>.

   NtimePt    The length of fullTime.

   Time       The part of fullTime contained in the time interval
              defined by the job input parameter Window <tcwt_help prm>.

   fTerp      The matrix of the transformation of the EEG from the time
              domain into the frequency domain; defined by Time and the job
              input parameters cutoffScale and fadeInOut <tcwt_help prm>.

   tTerp      The matrix of the transformation of the EEG from
              the frequency domain back into the time domain

   cwTerp     The matrix of the continuous wavelet transform (CWT) from
              the frequency domain onto a log-grid in the time-scale plane.

   logGrid    A two-row matrix of coordinates in the time-scale plane
              of the vertices of the CWT log-grid; defined by Time and
              job input parameters cutoffScale and logGridR <tcwt_help prm>.

 ALL JOB INPUT PARAMETERS ARE SAVED AS JOB CONSTANTS AS WELL.

Table of Contents
```

### prm2info

```
 tcwt_prm2info(prmFILE) displays the following values
 computed from the input parameter file prmFILE <tcwt_help prm>:

    T       Length of the time window ( = Window(2) - Window(1) )
    S       Cut-off scale ( = cutoffScale )
    R       Log-grid sampling rate ( = logGridR )

    NcwMax  Maximum number of trials processed by CWT in one step

    Nc      Number of channels to be processed by t-CWT
    Nf      Number of frequency components
    Nv      Number of log-grid vertexes

    Npca  = 2*(Nc*Nf)^2  Number of PCT and covariance matrix elements

    Ncwt  = Nc*Nf*Nv     Exact number of non-zero CWT matrix elements

    NcwtA = 12*Nc*((R*T)/S)^2   Approximate number of non-zero CWT ME

    Tpca    Estimated processing time for one PCA iteration
            assuming 1 microsecond per PCA matrix element

    Tcwt    Estimated processing time for one CWT scalogram
            assuming 1 microsecond per CWT matrix element

    Mpca    Estimated minimum amount of memory needed for PCA

    Mcwt    Estimated maximum amount of memory needed for CWT

 TIP: Try different parameter settings using the test parameter file
      tcwtROOT/jobs/test_prm.m' and typing "tcwt_prm2info test_prm".

Table of Contents
```

### dir2dsnl

```
 DSNL = tcwt_dir2dsnl(dsPATH,filenamePattern) creates a DataSet Name List
 (DSNL) from the output of a directory listing shell command executed
 in the directory specified by dsPATH with filenamePattern as a parameter.

 INPUT VARIABLES

   dsPATH             Full or relative path of the datasets' directory

   filenamePattern    File name pattern with wildcards ('*' and/or '?')

 OUTPUT VARIABLES

   DSNL       The DataSet Name List, a character array. Each row of DSNL
              is a DataSet Name (DSN). The DSNs are truncated names
              (basenames), i.e., dsPATH and all extensions are removed.

Table of Contents
```

### dsnl2xdsnl

```
 XDSNL = tcwt_dsnl2xdsnl(prefix,DSNL,suffix) creates an eXtended
 DataSet Name List (XDSNL) from an existing DataSet Name List
 (DSNL) <tcwt_help dir2dsnl> by adding a prefix to the beginning
 and a suffix to the end of each row (DSN) of DSNL.

 FUNCTION ARGUMENTS

   DSNL     An existing DataSet Name List

   prefix   The prexic added to each DSN

   suffix   The suffix added to each DSN

 OUTPUT VARIABLES

   XDSNL    The new eXtended DataSet Name List

Table of Contents
```

### ascii2tmat

```
 tcwt_ascii2tmat(DSNL,cndN) converts ASCII ERP to binary ERP.

 The job constants are loaded from './_A_const.mat' <tcwt_help prm2mat>.
 Then, the function iterates over DSNL reading and processing data from
 the INPUT FILES and saving results to the corresponding OUTPUT FILES.

 FUNCTION ARGUMENTS

   DSNL   DataSet Name List (DSN List) <tcwt_help dir2dsnl>

   cndN   A positive integer number denoting the experimental condition
          to which all trials in all ASCII files specified in DSNL belong.

 INPUT FILES               INPUT VARIABLES

   ../_ascii/DSN.ASCIIext   ASCII matrix, vectorized or multiplexed
                            (see below)

 ASCIIext is a job input parameter <tcwt_help prm>.

 OUTPUT FILES              OUTPUT VARIABLES (for details, see below)

   ../_tmat/DSN.t.mat       Verp,RIerp,CIerp

 INPUT ASCII DATA STRUCTURE

   Vectorized:

   v(1,1,1) v(1,1,2) v(1,1,3) ...  v(2,1,1) v(2,1,2) v(2,1,3) ...  ...
   v(1,2,1) v(1,2,2) v(1,2,3) ...  v(2,2,1) v(2,2,2) v(2,2,3) ...  ...
   v(1,3,1) v(1,3,2) v(1,3,3) ...  v(2,3,1) v(2,3,2) v(2,3,3) ...  ...
   ...
   ...
   ...

   Multiplexed:

   v(1,1,1)  v(1,2,1)  v(1,3,1) ...
   v(1,1,2)  v(1,2,2)  v(1,3,2) ...
   ...
   ...
   ...

   v(2,1,1)  v(2,2,1)  v(2,3,1) ...
   v(2,1,2)  v(2,2,2)  v(2,3,2) ...
   ...
   ...
   ...

   ...
   ...
   ...

 Notations used above:

   v(N,K,M)  Voltage value corresponding to the Nth trial,
             Kth channel, and Mth time point.

 The ASCII-to-binary conversion is performed according
 to the job input parameter isMultiplex <tcwt_help prm>.

 OUTPUT VARIABLES:     CIerp =

                       [ time(1)   time(2)   time(3)   ... ]

   RIerp =             Verp =

   [ 0 cndN 1 ;        [ v(1,1,1)  v(1,1,2)  v(1,1,3)  ... ;
     0 cndN 2 ;          v(1,2,1)  v(1,2,2)  v(1,2,3)  ... ;
     ...      ;          ...                               ;
     ...      ;          ...                               ;
     ...      ;          ...                               ;

     0 cndN 1 ;          v(2,1,1)  v(2,1,2)  v(2,1,3)  ... ;
     0 cndN 2 ;          v(2,2,1)  v(2,2,2)  v(2,2,3)  ... ;
     ...      ;          ...                               ;
     ...      ;          ...                               ;
     ...      ;          ...                               ;

     ...      ;          ...                               ;
     ...      ;          ...                               ;
     ...      ]          ...                               ]

 Notations used above:

   Verp      ERP matrix in the time domain

   CIerp     Verp column index

   RIerp     Verp row index

   v(N,K,M)  Voltage value corresponding to the Nth trial,
             Kth channel, and Mth time point.

   time(M)   Time (in seconds) corresponding to the Mth time point.

   cndN      Condition (experimental condition denoted
             by a number), to which all trials in this file belong.

Table of Contents
```

### t2f

```
 tcwt_t2f(DSNL) converts time domain ERP to frequency domain ERP.

 The job constants are loaded from './_A_const.mat' <tcwt_help prm2mat>.
 Then, the function iterates over DSNL reading and processing data from
 the INPUT FILES and saving results to the corresponding OUTPUT FILES.

 FUNCTION ARGUMENTS

   DSNL   DataSet Name List (DSN List) <tcwt_help dir2dsnl>

 INPUT FILES           INPUT VARIABLES

   ../_tmat/DSN.t.mat   Verp,RIerp,CIerp <tcwt_help ascii2tmat>

 OUTPUT FILES          OUTPUT VARIABLES (for details, see below)

   ./DSN.f.mat          Verp,CIerp
   ./DSN.ri0.mat        RIerp,DSNLpool (DSNLpool=DSN) <tcwt_help f2pool>

 The following steps precede the frequency domain transformation:

   Channel selection:  Channels that are not used according to the entries
                       in ChannelList <tcwt_help prm> are deleted.

   Baseline reference: BaseLine is a job input parameter <tcwt_help prm>.

   Window selection:   Data points outside of the time interval defined
                       by the job input parameter Window are deleted.

 The transform from the time domain to the frequency domain is performed
 by multiplication with the t-CWT CONSTANT fTerp <tcwt_help prm2mat>.
 It comprises time domain multiplication with a Tukey window function
 defined by the job input parameter fadeInOut, Discrete Fourier Transform
 (DFT), and low-pass filtering with a cutoff frequency = 1/cutoffScale
 <tcwt_help prm>. For math details: <tcwt_help math>.

 OUTPUT VARIABLES:     CIerp =

                       [ chnum(1) chnum(1)...  chnum(2) chnum(2)...  ... ]

   RIerp =             Verp =

   [ out(1) cndN ;     [ v(1,1,1) v(1,1,2)...  v(1,2,1) v(1,2,2)...  ... ;
     out(2) cndN ;       v(2,1,1) v(2,1,2)...  v(2,2,1) v(2,2,2)...  ... ;
     out(3) cndN ;       v(3,1,1) v(3,1,2)...  v(3,2,1) v(3,2,2)...  ... ;
     ...         ;       ...                                             ;
     ...         ;       ...                                             ;
     ...         ]       ...                                             ]

 Notations used above:

   Verp      ERP matrix in the frequency domain

   CIerp     Verp column index

   RIerp     Verp row index

   v(N,K,M)  Value corresponding to the Nth trial,
             Kth channel, and Mth frequency.

   chnum(K)  Channel number of the Kth channel.

   out(N)    Outlier mark (0 or 1) of the Nth trial (1 = marked as outlier).

   cndN      Condition (experimental condition denoted
             by a number), to which all trials of this dataset belong.

Table of Contents
```

### f2merge

```
 tcwt_f2merge(DSNL,suffixLIST,mergeSFX) merges two or more ERP datasets.

 The function iterates over DSNL and suffixLIST reading data from the
 INPUT FILES and saving merged data to the corresponding OUTPUT FILES.

 FUNCTION ARGUMENTS

   DSNL         DataSet Name List (DSN List) <tcwt_help dir2dsnl>

   suffixLIST = [sfx1; sfx2; ... ] Suffix List of datasets to be merged.

   mergeSFX     The suffix of the resulting merger dataset.

 INPUT FILES          INPUT VARIABLES <tcwt_help t2f>

   ./DSNsfx1.f.mat     Verp = Verp1, CIerp
   ./DSNsfx2.f.mat     Verp = Verp2, CIerp
   ...                 ...

   ./DSNsfx1.ri0.mat   RIerp = RIerp1, DSNLpool
   ./DSNsfx2.ri0.mat   RIerp = RIerp2, DSNLpool
   ...                 ...

 OUTPUT FILES             OUTPUT VARIABLES

   ./DSNmergeSFX.f.mat     Verp = [Verp1; Verp2; ... ]
                           CIerp

   ./DSNmergeSFX.ri0.mat   RIerp = [RIerp1; RIerp2; ... ]
                           DSNLpool = [DSN mergeSFX] <tcwt_help f2pool>

Table of Contents
```

### f2split

```
 tcwt_f2split(DSNL,trialrangeLIST,suffixLIST) splits ERP datasets
                                              into smaller subsets.

 The function iterates over DSNL reading data from the INPUT FILES,
 splitting it according to trialrangeLIST, and saving the split data
 to the corresponding OUTPUT FILES according to suffixLIST.

 FUNCTION ARGUMENTS

   DSNL             DataSet Name List (DSN List) <tcwt_help dir2dsnl>

   trialrangeLIST = [first1 last1; first2 last2;  ... ]   A list of
                    trial intervals defining the subsets into which
                    each dataset DSN will be split

   suffixLIST   =   [sfx1; sfx2; ... ]    A list of suffixes
                    corresponding to the trial intervals defined
                    by trialrangeLIST to be added to each DSN to
                    name the corresponding subsets of ERP trails

 INPUT FILES            INPUT VARIABLES <tcwt_help t2f>

   ./DSN.f.mat           Verp,CIerp
   ./DSN.ri0.mat         RIerp,DSNLpool

 OUTPUT FILES           OUTPUT VARIABLES

   ./DSNsfx1.f.mat       Verp,CIerp
   ./DSNsfx2.f.mat       Verp,CIerp
   ...                   ...

   ./DSNsfx1.ri0.mat     RIerp,DSNLpool
   ./DSNsfx2.ri0.mat     RIerp,DSNLpool
   ...                   ...

Table of Contents
```

### f2pool

```
 tcwt_f2pool(DSNL,poolNAME) pools two or more individual ERP datasets
                            together into one large group dataset.

 The job constants are loaded from './_A_const.mat' <tcwt_help prm2mat>.
 Then, the function iterates over DSNL reading data from the INPUT FILES
 and saving the pooled data to the corresponding OUTPUT FILES.

 FUNCTION ARGUMENTS

   DSNL      DataSet Name List (DSN List) <tcwt_help dir2dsnl>

   poolNAME  The Name of the pooled dataset

 INPUT FILES       INPUT VARIABLES <tcwt_help t2f>

   ./DSN1.f.mat     Verp1,CIerp1
   ./DSN2.f.mat     Verp2,CIerp2
   ...                 ...

   ./DSN1.ri0.mat   RIerp1,DSNLpool
   ./DSN2.ri0.mat   RIerp2
   ...                 ...

 OUTPUT FILES          OUTPUT VARIABLES

   ./poolNAME.f.mat     Verp = [Verp1; Verp2; ... ]
                        CIerp = CIerp1

   ./poolNAME.ri0.mat   RIerp = [RIerp1; RIerp2; ... ]
                        DSNLpool = DSNL

Table of Contents
```

### f2pc

```
 tcwt_f2pc(DSNL,Npc,Nstd,MinGood) Principal Component Transform (PCT),
 Principal Component Analysis (PCA), and Multivariate Outlier Detection.

 The function iterates over DSNL reading and processing data from
 the INPUT FILES and saving results to the corresponding OUTPUT FILES.

 FUNCTION ARGUMENTS

   DSNL     DataSet Name List (DSN List) <tcwt_help dir2dsnl>

   Npc      VALUE      defines   PCA CRITERION <tcwt_help math>

             Npc > 1              the first Npc eigenvalues will be retained
             Npc = 1              all eigenvalues will be retained
             0 < Npc < 1          percentage of variance to be explained
             Npc = 0              average eigenvalue criterion

   Nstd > 2 Number of standard deviations for the single-trial outlier
            detection criterion: D > Mean(D) + Nstd * StdDev(D), where
            D = single-trial Mahalanobis Distance from mean <tcwt_help math>

   MinGood  Defines the dataset outlier detection criterion <tcwt_help math>:
            number_of_good_trials < MinGood * number_of_trials_in_dataset

 INPUT FILES      INPUT VARIABLES <tcwt_help t2f>

   ./DSN.f.mat     Verp,CIerp
   ./DSN.ri0.mat   RIerp, DSNLpool <tcwt_help f2pool>

 OUTPUT FILES     OUTPUT VARIABLES

   ./DSN.pc.mat    pcTerp,pcEV
   ./DSN.ri1.mat   RIerp, DSNLpool
   ./DSN.ri1.txt   Text output

 OUTPUT VARIABLES

   pcTerp   Reduced PCT matrix. <tcwt_help math>. The principal components
            pcVerp of the ERP sample Verp are: pcVerp = Verp * pcTerp

   pcEV     Reduced PCT eigenvalues <tcwt_help math>

   RIerp    ERP Row Index; 1st row contains outlier marks <tcwt_help t2f>

Table of Contents
```

### f2cwss

```
 tcwt_f2cwss(DSNL) computes the Sums of Squares (SS) of the Continuous
 Wavelet Transform (CWT) for subsequent Student's t-tests <tcwt_help math>.

 The job constants are loaded from './_A_const.mat' <tcwt_help prm2mat>.
 Then, the function iterates over DSNL reading and processing data from
 the INPUT FILES and saving results to the corresponding OUTPUT FILES.

 FUNCTION ARGUMENTS

   DSNL   DataSet Name List (DSN List) <tcwt_help dir2dsnl>

 INPUT FILES         INPUT VARIABLES

   ./DSN.f.mat        Verp,CIerp <tcwt_help t2f>
   ./DSN.pc.mat       pcTerp <tcwt_help f2pc>
   ./DSN.ri1.mat      RIerp <tcwt_help t2f>

 OUTPUT FILES        OUTPUT VARIABLES

   ./DSN.cwss.mat        cwVerpSS

Table of Contents
```

### ri2ri1out

```
 tcwt_ri2ri1out(poolNAME) creates 1out-indexes from the row index
 of a pooled dataset excluding one individual dataset at a time.

 FUNCTION ARGUMENTS

   poolNAME  The Name of the pooled dataset

 INPUT FILE                      INPUT VARIABLES

   ./poolNAME.ri1.mat              RIerp,DSNLpool <tcwt_help f2pool>

 OUTPUT FILES                    OUTPUT VARIABLES

   ./DSNp.out.poolNAME.ri1.mat     RIerp,DSNLpool

 DSNp is taken from DSNLpool.

 The function iterates over DSNLpool. At each iteration, DSNp is read
 from the next row of DSNLpool and all trials belonging to the dataset
 DSNp are marked as artifacts. RIerp, thus modified, is stored in the 
 corresponding OUTPUT FILE.

Table of Contents
```

### pc2cnd2ri

```
 tcwt_pc2cnd2ri(is1out,DSNL,Cnd,Nstd,MinGood) Multivariate Outlier Detection
 from fixed principal components, separately for each experimental condition.

 The function iterates over DSNL reading and processing data from
 the INPUT FILES and saving results to the corresponding OUTPUT FILES.
 If is1out = 1, a second DSNLpool of 1out index names <tcwt_help ri2ri1out>
 is created at each step, and the function iterates throuh DSNLpool.

 FUNCTION ARGUMENTS

   is1out = 0 or 1; defines whether 1out indexes are used (see below)

   DSNL     DataSet Name List (DSN List) <tcwt_help dir2dsnl>

   Cnd      List of experimental conditions <tcwt_help t2f>

   Nstd     <tcwt_help f2pc>

   MinGood  <tcwt_help f2pc>

 INPUT FILES               INPUT VARIABLES

   ./DSN.f.mat              Verp,CIerp <tcwt_help t2f>
   ./DSN.pc.mat             pcTerp,pcEV <tcwt_help f2pc>
   ./DSN.ri1.mat            RIerp, DSNLpool <tcwt_help f2pool>

  If is1out == 1

   ./DSNp.out.DSN.ri1.mat   RIerp

 DSNp is taken from DSNLpool.

 OUTPUT FILES              OUTPUT VARIABLES

   ./DSN.ri2.mat            RIerp, DSNLpool
   ./DSN.ri2.txt            Text output

   If is1out == 1

   ./DSNp.out.DSN.ri2.mat   RIerp, DSNLpool
   ./DSNp.1out.ri2.mat      Text output

Table of Contents
```

### f2x

```
 tcwt_f2x(is1out,DSNL,Cnd) t-CWT & Extremum Detection <tcwt_help math>.

 The job constants are loaded from './_A_const.mat' <tcwt_help prm2mat>.
 Then, the function iterates over DSNL reading and processing data from
 the INPUT FILES and saving results to the corresponding OUTPUT FILES.
 If is1out = 1, a second DSNLpool of 1out index names <tcwt_help ri2ri1out>
 is created at each step, and the function iterates throuh DSNLpool.

 FUNCTION ARGUMENTS

   is1out = 0 or 1; defines whether 1out indexes are used (see below)

   DSNL     DataSet Name List (DSN List) <tcwt_help dir2dsnl>

   Cnd      List of exactly 2 experimental conditions <tcwt_help t2f>

 INPUT FILES               INPUT VARIABLES

   ./DSN.f.mat              Verp,CIerp <tcwt_help t2f>
   ./DSN.pc.mat             pcTerp,pcEV <tcwt_help f2pc>
   ./DSN.cwss.mat           cwVerpSS <tcwt_help f2cwss>
   ./DSN.ri1.mat            RIerp, DSNLpool <tcwt_help f2pool>
   ./DSN.ri2.mat            RIerp, DSNLpool

  If is1out == 1

   ./DSNp.out.DSN.ri2.mat   RIerp

 DSNp is taken from DSNLpool.

 OUTPUT FILES              OUTPUT VARIABLES

   ./DSN.tcw.mat            tcwVerp,tcwCIerp
   ./DSN.x.mat              xTerp,xCIerp

   If is1out == 1

   ./DSNp.out.DSN.tcw.mat   tcwVerp,tcwCIerp
   ./DSNp.out.DSN.x.mat     xTerp,xCIerp

 OUTPUT VARIABLES

   tcwCIerp =

   [ chnum(1) chnum(1)... chnum(1) chnum(1)... chnum(2) chnum(2)... ... ;
     scale(1) scale(1)... scale(2) scale(2)... scale(1) scale(1)... ... ;
     time(1,1)time(1,2)...time(2,1)time(2,2)...time(1,1)time(1,2)... ... ]

   tcwVerp =

   [ t(1,1,1) t(1,1,2)... t(1,2,1) t(1,2,2)... t(2,1,1) t(2,1,2)... ... ]

   xCIerp =

   [ xchnum(1)    ... xchnum(2)    ...  ... ;
     xscale(1)    ... xscale(2)    ...  ... ;
     xtime(1)     ... xtime(2)     ...  ... ;
     xpolarity(1) ... xpolarity(2) ...  ... ;
     xtcwindex(1) ... xtcwindex(2) ...  ... ;
     xtcwvalue(1) ... xtcwvalue(2) ...  ... ]

 Notations used above:

   tcwVerp    t-CWT scalogram

   tcwCIerp   t-CWT column index

   xTerp      Transformation matrix from the frequency domain ERP Verp
              to the t-CWT features (extrema) xVerp = Verp * xTerp

   xCIerp     t-CWT extrema (i.e. xVerp column index)

   t(K,N,M)   t-CWT-value corresponding to the Kth channel, and the Mth
              time point of the Nth scale line of logGrid <tcwt_help prm2mat>

   chnum(K)   Channel number of the Kth channel

   scale(N)   Scale in seconds of the Nth scale line of logGrid

   time(N,M)  Time in seconds of the Mth time point of the Nth scale line

   xchnum(I)      Channel number of the Ith extremum

   xscale(I)      Scale in seconds of the Ith extremum

   xtime(I)       Time in seconds of the Ith extremum

   xpolarity(I)   Polarity of the Ith extremum (-1 = min, 1 = max)

   xtcwindex(I)   Absolute tcwVerp index of the Ith extremum

   xtcwvalue(I) = tcwVerp( xtcwindex(I) )

Table of Contents
```

### x2ld

```
 tcwt_x2ld(is1out,DSNL,Cnd,Npc,sdAlpha) Principal Component Analysis
 (PCA), from the t-CWT features, Step-Down Selection of components, and
 computation of the Linear Discriminant Function (LDF) <tcwt_help math>

 The function iterates over DSNL reading and processing data from
 the INPUT FILES and saving results to the corresponding OUTPUT FILES.
 If is1out = 1, a second DSNLpool of 1out index names <tcwt_help ri2ri1out>
 is created at each step, and the function iterates throuh DSNLpool.

 FUNCTION ARGUMENTS

   is1out = 0 or 1; defines whether 1out indexes are used (see below)

   DSNL     DataSet Name List (DSN List) <tcwt_help dir2dsnl>

   Cnd      List of exactly 2 experimental conditions <tcwt_help t2f>

   Npc      PCA criterion for the step-down test <tcwt_help f2pc>

   sdAlpha  Overall alpha level for the step-down test <tcwt_help math>

 INPUT FILES               INPUT VARIABLES

   ./DSN.f.mat              Verp,CIerp <tcwt_help t2f>
   ./DSN.x.mat              xCIerp,xTerp <tcwt_help f2x>
   ./DSN.ri1.mat            RIerp, DSNLpool <tcwt_help f2pool>
   ./DSN.ri2.mat            RIerp, DSNLpool

  If is1out == 1

   ./DSNp.out.DSN.x.mat     xCIerp,xTerp
   ./DSNp.out.DSN.ri2.mat   RIerp

 DSNp is taken from DSNLpool.

 OUTPUT FILES              OUTPUT VARIABLES

   ./DSN.ld.mat             Nxtr,sdTerp,ldTerp,ldMean,CIerp

   If is1out == 1

   ./DSNp.out.DSN.ld.mat    Nxtr,sdTerp,ldTerp,ldMean,CIerp

 OUTPUT VARIABLES

   Nxtr     The number of t-CWT extrema (features)

   sdTerp   Transformation matrix from the frequency domain ERP Verp
            to the principal components obtained from the t-CWT features
            and reduced by step-down selection sdVerp = Verp * sdTerp

   ldTerp   Frequency domain LDF obtained from sdVerp. The scalar product
            with the frequency domain ERP Verp is simply: Verp * ldTerp

   ldMean = (1/2) * ( mean(Verp1) + mean(Verp2) ) * ldTerp
            Verp1 and Verp2 are the ERP samples corresponding to the
            experimental conditions Cnd(1) and Cnd(2), respectively.

   CIerp    ERP column index in the frequency domain <tcwt_help t2f>

Table of Contents
```

### f2stats

```
 tcwt_f2stats(is1out,DSNL1,DSNL2,statsNAME,Cnd,aPriori) performs
 LDA classification of single trials from test datasets by LDFs
 obtained from training datasets <tcwt_help x2ld>.

 The function iterates over DSNL2 reading and processing data from
 the INPUT FILES and then saves the results into the OUTPUT FILES.

 FUNCTION ARGUMENTS

   is1out  =  0 or 1; defines whether 1out indexes are used (see below)

   DSNL1      Training DataSet Name List (DSN1 List) <tcwt_help dir2dsnl>

   DSNL2      Test DataSet Name List (DSN2 List)

   statsNAME  Name of the OUTPUT FILES (without the extensions)

   Cnd        List of exactly 2 experimental conditions <tcwt_help t2f>

   aPriori  = 0 or 1; defines whether the a priori error rate is computed
              from the numbers of trials in Cnd(1) and Cnd(2).
              If aPriori == 0, the a priori error rate and the a priori
              hit rate are taken to be equal (p1=p2=0.5) and log(p2/p1)=0.

 INPUT FILES              INPUT VARIABLES

   ./DSN2.f.mat            Verp,CIerp <tcwt_help t2f>
   ./DSN2.ri0.mat          RIerp, DSNLpool <tcwt_help f2pool>

  If is1out == 0

   ./DSN1.ld.mat           Nxtr,sdTerp,ldTerp,ldMean,CIerp <tcwt_help x2ld>

  If is1out == 1, and DSNL1 has only one row, DSN1

   ./DSN2.out.DSN1.ld.mat  Nxtr,sdTerp,ldTerp,ldMean,CIerp <tcwt_help x2ld>

 OUTPUT FILES                 OUTPUT VARIABLES

   ./statsNAME.stats0.mat      dsnl = DSNL2,
      (if aPriori == 0)        NtCWTextr,NsdPC,Photelling
   ./statsNAME.statsA.mat      Ntrials,Nerrors,ErrP100,Pbinomi
      (if aPriori == 1)        NtrialsSum,NerrorsSum,ErrP100Sum,PbinomiSum

   ./statsNAME.stats0.txt      Text output
   ./statsNAME.statsA.txt

  If DSNL1 == DSNL2

   ./statsNAME.biased.stats0.mat
   ./statsNAME.biased.stats0.txt

   ./statsNAME.biased.statsA.mat
   ./statsNAME.biased.statsA.txt

  If is1out == 1, and DSNL1 has only one row

   ./statsNAME.1dataset.out.stats0.mat
   ./statsNAME.1dataset.out.stats0.txt

   ./statsNAME.1dataset.out.statsA.mat
   ./statsNAME.1dataset.out.statsA.txt

 OUTPUT VARIABLES

   NtCWTextr    Number of t-CWT features for each training dataset
                (DSN1.ld.mat or DSN2.out.DSN1.ld.mat, see INPUT FILES)

   NsdPC        Number of step-down-selected components
                in each training dataset

   Photelling   P-Value of Hotelling's test performed on each test
                dataset DSN2 using the step-down-selected components
                from the corresponding training dataset

   Ntrials      Nubmer of trials, for each condition and total,
                in each DSN2. Ntrials(:,1) corresponds to Cnd(1)
                Ntrials(:,2) corresponds to Cnd(2), and Ntrials(:,3)
                is the total nubmer of trials

   Nerrors      Number of errors of LDA classification, for Cnd(1), Cnd(2),
                and total, for each DSN2. Cnd(1) --> Nerrors(:,1), etc.

   ErrP100  =   100 * Nerrors / Ntrials    (error percentages)

   Pbinomi      P-Values of the binomial distribution testing the hypotheses
                that the LDA classifications were better than chance,
                i.e., that ErrP100 < 50%

   NtrialsSum = sum(Ntrials)

   NerrorsSum = sum(Nerrors)

   ErrP100Sum = 100 * NerrorsSum / NtrialsSum   (average error percentages)

   PbinomiSum   P-Values for testing whether ErrP100Sum < 50%

Table of Contents
```

### f2holdout

```
 tcwt_f2holdout(DSNL,statsNAME,Cnd,Nstd,MinGood,Npc,sdAlpha) computes
 the error rates of LDA classificaion of single trials using the
 hold-out method <tcwt_help math>.

 The function iterates over DSNL reading and processing data from
 the INPUT FILES and then saves the results into the OUTPUT FILES.

 For each DSN, the hold-out method is applied by iterating through all
 trials of the dataset, excluding the current trial, doing t-CWT with
 the other trials <tcwt_help pc2ri2cnd> <tcwt_help f2x> <tcwt_help x2ld>
 and applying the obtained LDF to the current trial <tcwt_help math>.
 Classification errors and binomial p-values are computed and saved
 in the OUTPUT FILES <tcwt_help stats>.

 FUNCTION ARGUMENTS

   DSNL       Test DataSet Name List (DSN List) <tcwt_help dir2dsnl>

   statsNAME  Name of the OUTPUT FILES (without the extensions)

   Cnd        List of exactly 2 experimental conditions <tcwt_help t2f>

   Nstd       Criterion for single-trial outlier detection <tcwt_help f2pc>

   MinGood    Criterion for dataset outlier detection <tcwt_help f2pc>

   Npc        PCA criterion for the step-down test <tcwt_help f2pc>

   sdAlpha    Overall alpha level for the step-down test <tcwt_help math>

 INPUT FILES               INPUT VARIABLES

   ./DSN.f.mat              Verp,CIerp <tcwt_help t2f>
   ./DSN.pc.mat             pcTerp,pcEV <tcwt_help f2pc>
   ./DSN.cwss.mat           cwVerpSS <tcwt_help f2cwss>
   ./DSN.ri0.mat            RIerp, DSNLpool <tcwt_help f2pool>
   ./DSN.ri1.mat            RIerp, DSNLpool

 OUTPUT FILES                          OUTPUT VARIABLES <tcwt_help f2stats>

   ./statsNAME.1trial.out.stats0.mat    dsnl = DSNL,
   ./statsNAME.1trial.out.statsA.mat    Ntrials,Nerrors,ErrP100,Pbinomi
                                        NtrialsSum,NerrorsSum,ErrP100Sum,
                                        PbinomiSum

   ./statsNAME.1trial.out.stats0A.txt   Text output

   Suffix '0' denotes that the priori probabilities are taken to be equal;
   'A' means they are computed; from the number of trials in each condition.

Table of Contents
```

### f2plot

```
 tcwt_f2plot(DSNL,cxyFILE,Cnd,CndWidth,CndRGB,tTestWidth,tTestRGB) plots
 two average ERPs and Student's t-test of the difference ERP.

 The job constants are loaded from './_A_const.mat' <tcwt_help prm2mat>.
 Then, the function iterates over DSNL reading data from the INPUT FILES,
 computing ERP averages corresponding to two different experimental
 conditions and Student's t-test of the difference ERP, plotting the
 results, and saving the plots to the corresponding OUTPUT PDF FILES.

 FUNCTION ARGUMENTS

   DSNL        DataSet Name List (DSN List) <tcwt_help dir2dsnl>

   cxyFILE     The basename (without the path and the '.m' extension)
               of the file, containing the X-Y-coordinates of
               the channels in the subplot array <tcwt_help cxy>.

   Cnd      =  [cnd1; cnd2]   Experimental conditions <tcwt_help t2f>

   CndWidth =  [width1; width2]   Line widths of the average ERP curves
               corresponding to cnd1 and cnd2 respectively

   CndRGB   =  [R1 G1 B1; R2 G2 B2]  Line colors (defined as RGB vectors) of
               the average ERPs corresponding to cnd1 and cnd2 respectively

   tTestWidth  Line width of the t-test curve

   tTestRGB    Line color (defined as an RGB vector) of the t-test curve

 INPUT FILES               INPUT VARIABLES

   ./DSN.f.mat               Verp,CIerp <tcwt_help t2f>
   ./DSN.pc.mat              pcTerp <tcwt_help f2pc>
   ./DSN.ri2.mat             RIerp <tcwt_help t2f>

 OUTPUT FILES              OUTPUT VARIABLES

   ./DSN.plot.avg.jpg        average ERP plot as JPG
   ./DSN.plot.ttest.jpg      Student's t-test plot as JPG

 Note that ERP averaging is done after PCA filtering and double outlier
 rejection: first, regardless of experimental conditions, and second,
 performed on each condition separately. The t-test is also done with
 the corresponding ERP subsamples, which means that it is biased and
 should be used only for visualization purposes.

Table of Contents
```

### ld2plot

```
 tcwt_ld2plot(DSNL,cxyFILE,normC,LineWidth,ColorRGB) plots the normalized
 Linear Discriminant Function (LDF).

 The job constants are loaded from './_A_const.mat' <tcwt_help prm2mat>.
 Then, the function iterates over DSNL plotting the Linear Discriminant
 Functions (LDFs) <tcwt_help x2ld> stored in the INPUT FILES and saving
 the plots to the corresponding OUTPUT PDF FILES.

 FUNCTION ARGUMENTS

   DSNL        DataSet Name List (DSN List) <tcwt_help dir2dsnl>

   cxyFILE     The basename (without the path and the '.m' extension)
               of the file, containing the X-Y-coordinates of
               the channels in the subplot array <tcwt_help cxy>.

   normC       A coefficient with which the LDF is multiplied
               for better visualization (can be negative).

   LineWidth   The line width

   ColorRGB  = [R, G, B]   The line color defined as an RGB vector

 INPUT FILES          INPUT VARIABLES

   ./DSN.ld.mat         Nxtr,sdTerp,ldTerp,ldMean,CIerp <tcwt_help x2ld>

 OUTPUT FILES         OUTPUT VARIABLES

   ./DSN.plot.ld.jpg   ldTerp plot as JPG

Table of Contents
```

### gpl

```
                     GNU GENERAL PUBLIC LICENSE
                        Version 3, 29 June 2007
 
  Copyright (C) 2007 Free Software Foundation, Inc. <http://fsf.org/>
  Everyone is permitted to copy and distribute verbatim copies
  of this license document, but changing it is not allowed.
 
                             Preamble
 
   The GNU General Public License is a free, copyleft license for
 software and other kinds of works.
 
   The licenses for most software and other practical works are designed
 to take away your freedom to share and change the works.  By contrast,
 the GNU General Public License is intended to guarantee your freedom to
 share and change all versions of a program--to make sure it remains free
 software for all its users.  We, the Free Software Foundation, use the
 GNU General Public License for most of our software; it applies also to
 any other work released this way by its authors.  You can apply it to
 your programs, too.
 
   When we speak of free software, we are referring to freedom, not
 price.  Our General Public Licenses are designed to make sure that you
 have the freedom to distribute copies of free software (and charge for
 them if you wish), that you receive source code or can get it if you
 want it, that you can change the software or use pieces of it in new
 free programs, and that you know you can do these things.
 
   To protect your rights, we need to prevent others from denying you
 these rights or asking you to surrender the rights.  Therefore, you have
 certain responsibilities if you distribute copies of the software, or if
 you modify it: responsibilities to respect the freedom of others.
 
   For example, if you distribute copies of such a program, whether
 gratis or for a fee, you must pass on to the recipients the same
 freedoms that you received.  You must make sure that they, too, receive
 or can get the source code.  And you must show them these terms so they
 know their rights.
 
   Developers that use the GNU GPL protect your rights with two steps:
 (1) assert copyright on the software, and (2) offer you this License
 giving you legal permission to copy, distribute and/or modify it.
 
   For the developers' and authors' protection, the GPL clearly explains
 that there is no warranty for this free software.  For both users' and
 authors' sake, the GPL requires that modified versions be marked as
 changed, so that their problems will not be attributed erroneously to
 authors of previous versions.
 
   Some devices are designed to deny users access to install or run
 modified versions of the software inside them, although the manufacturer
 can do so.  This is fundamentally incompatible with the aim of
 protecting users' freedom to change the software.  The systematic
 pattern of such abuse occurs in the area of products for individuals to
 use, which is precisely where it is most unacceptable.  Therefore, we
 have designed this version of the GPL to prohibit the practice for those
 products.  If such problems arise substantially in other domains, we
 stand ready to extend this provision to those domains in future versions
 of the GPL, as needed to protect the freedom of users.
 
   Finally, every program is threatened constantly by software patents.
 States should not allow patents to restrict development and use of
 software on general-purpose computers, but in those that do, we wish to
 avoid the special danger that patents applied to a free program could
 make it effectively proprietary.  To prevent this, the GPL assures that
 patents cannot be used to render the program non-free.
 
   The precise terms and conditions for copying, distribution and
 modification follow.
 
                        TERMS AND CONDITIONS
 
   0. Definitions.
 
   "This License" refers to version 3 of the GNU General Public License.
 
   "Copyright" also means copyright-like laws that apply to other kinds of
 works, such as semiconductor masks.
 
   "The Program" refers to any copyrightable work licensed under this
 License.  Each licensee is addressed as "you".  "Licensees" and
 "recipients" may be individuals or organizations.
 
   To "modify" a work means to copy from or adapt all or part of the work
 in a fashion requiring copyright permission, other than the making of an
 exact copy.  The resulting work is called a "modified version" of the
 earlier work or a work "based on" the earlier work.
 
   A "covered work" means either the unmodified Program or a work based
 on the Program.
 
   To "propagate" a work means to do anything with it that, without
 permission, would make you directly or secondarily liable for
 infringement under applicable copyright law, except executing it on a
 computer or modifying a private copy.  Propagation includes copying,
 distribution (with or without modification), making available to the
 public, and in some countries other activities as well.
 
   To "convey" a work means any kind of propagation that enables other
 parties to make or receive copies.  Mere interaction with a user through
 a computer network, with no transfer of a copy, is not conveying.
 
   An interactive user interface displays "Appropriate Legal Notices"
 to the extent that it includes a convenient and prominently visible
 feature that (1) displays an appropriate copyright notice, and (2)
 tells the user that there is no warranty for the work (except to the
 extent that warranties are provided), that licensees may convey the
 work under this License, and how to view a copy of this License.  If
 the interface presents a list of user commands or options, such as a
 menu, a prominent item in the list meets this criterion.
 
   1. Source Code.
 
   The "source code" for a work means the preferred form of the work
 for making modifications to it.  "Object code" means any non-source
 form of a work.
 
   A "Standard Interface" means an interface that either is an official
 standard defined by a recognized standards body, or, in the case of
 interfaces specified for a particular programming language, one that
 is widely used among developers working in that language.
 
   The "System Libraries" of an executable work include anything, other
 than the work as a whole, that (a) is included in the normal form of
 packaging a Major Component, but which is not part of that Major
 Component, and (b) serves only to enable use of the work with that
 Major Component, or to implement a Standard Interface for which an
 implementation is available to the public in source code form.  A
 "Major Component", in this context, means a major essential component
 (kernel, window system, and so on) of the specific operating system
 (if any) on which the executable work runs, or a compiler used to
 produce the work, or an object code interpreter used to run it.
 
   The "Corresponding Source" for a work in object code form means all
 the source code needed to generate, install, and (for an executable
 work) run the object code and to modify the work, including scripts to
 control those activities.  However, it does not include the work's
 System Libraries, or general-purpose tools or generally available free
 programs which are used unmodified in performing those activities but
 which are not part of the work.  For example, Corresponding Source
 includes interface definition files associated with source files for
 the work, and the source code for shared libraries and dynamically
 linked subprograms that the work is specifically designed to require,
 such as by intimate data communication or control flow between those
 subprograms and other parts of the work.
 
   The Corresponding Source need not include anything that users
 can regenerate automatically from other parts of the Corresponding
 Source.
 
   The Corresponding Source for a work in source code form is that
 same work.
 
   2. Basic Permissions.
 
   All rights granted under this License are granted for the term of
 copyright on the Program, and are irrevocable provided the stated
 conditions are met.  This License explicitly affirms your unlimited
 permission to run the unmodified Program.  The output from running a
 covered work is covered by this License only if the output, given its
 content, constitutes a covered work.  This License acknowledges your
 rights of fair use or other equivalent, as provided by copyright law.
 
   You may make, run and propagate covered works that you do not
 convey, without conditions so long as your license otherwise remains
 in force.  You may convey covered works to others for the sole purpose
 of having them make modifications exclusively for you, or provide you
 with facilities for running those works, provided that you comply with
 the terms of this License in conveying all material for which you do
 not control copyright.  Those thus making or running the covered works
 for you must do so exclusively on your behalf, under your direction
 and control, on terms that prohibit them from making any copies of
 your copyrighted material outside their relationship with you.
 
   Conveying under any other circumstances is permitted solely under
 the conditions stated below.  Sublicensing is not allowed; section 10
 makes it unnecessary.
 
   3. Protecting Users' Legal Rights From Anti-Circumvention Law.
 
   No covered work shall be deemed part of an effective technological
 measure under any applicable law fulfilling obligations under article
 11 of the WIPO copyright treaty adopted on 20 December 1996, or
 similar laws prohibiting or restricting circumvention of such
 measures.
 
   When you convey a covered work, you waive any legal power to forbid
 circumvention of technological measures to the extent such circumvention
 is effected by exercising rights under this License with respect to
 the covered work, and you disclaim any intention to limit operation or
 modification of the work as a means of enforcing, against the work's
 users, your or third parties' legal rights to forbid circumvention of
 technological measures.
 
   4. Conveying Verbatim Copies.
 
   You may convey verbatim copies of the Program's source code as you
 receive it, in any medium, provided that you conspicuously and
 appropriately publish on each copy an appropriate copyright notice;
 keep intact all notices stating that this License and any
 non-permissive terms added in accord with section 7 apply to the code;
 keep intact all notices of the absence of any warranty; and give all
 recipients a copy of this License along with the Program.
 
   You may charge any price or no price for each copy that you convey,
 and you may offer support or warranty protection for a fee.
 
   5. Conveying Modified Source Versions.
 
   You may convey a work based on the Program, or the modifications to
 produce it from the Program, in the form of source code under the
 terms of section 4, provided that you also meet all of these conditions:
 
     a) The work must carry prominent notices stating that you modified
     it, and giving a relevant date.
 
     b) The work must carry prominent notices stating that it is
     released under this License and any conditions added under section
     7.  This requirement modifies the requirement in section 4 to
     "keep intact all notices".
 
     c) You must license the entire work, as a whole, under this
     License to anyone who comes into possession of a copy.  This
     License will therefore apply, along with any applicable section 7
     additional terms, to the whole of the work, and all its parts,
     regardless of how they are packaged.  This License gives no
     permission to license the work in any other way, but it does not
     invalidate such permission if you have separately received it.
 
     d) If the work has interactive user interfaces, each must display
     Appropriate Legal Notices; however, if the Program has interactive
     interfaces that do not display Appropriate Legal Notices, your
     work need not make them do so.
 
   A compilation of a covered work with other separate and independent
 works, which are not by their nature extensions of the covered work,
 and which are not combined with it such as to form a larger program,
 in or on a volume of a storage or distribution medium, is called an
 "aggregate" if the compilation and its resulting copyright are not
 used to limit the access or legal rights of the compilation's users
 beyond what the individual works permit.  Inclusion of a covered work
 in an aggregate does not cause this License to apply to the other
 parts of the aggregate.
 
   6. Conveying Non-Source Forms.
 
   You may convey a covered work in object code form under the terms
 of sections 4 and 5, provided that you also convey the
 machine-readable Corresponding Source under the terms of this License,
 in one of these ways:
 
     a) Convey the object code in, or embodied in, a physical product
     (including a physical distribution medium), accompanied by the
     Corresponding Source fixed on a durable physical medium
     customarily used for software interchange.
 
     b) Convey the object code in, or embodied in, a physical product
     (including a physical distribution medium), accompanied by a
     written offer, valid for at least three years and valid for as
     long as you offer spare parts or customer support for that product
     model, to give anyone who possesses the object code either (1) a
     copy of the Corresponding Source for all the software in the
     product that is covered by this License, on a durable physical
     medium customarily used for software interchange, for a price no
     more than your reasonable cost of physically performing this
     conveying of source, or (2) access to copy the
     Corresponding Source from a network server at no charge.
 
     c) Convey individual copies of the object code with a copy of the
     written offer to provide the Corresponding Source.  This
     alternative is allowed only occasionally and noncommercially, and
     only if you received the object code with such an offer, in accord
     with subsection 6b.
 
     d) Convey the object code by offering access from a designated
     place (gratis or for a charge), and offer equivalent access to the
     Corresponding Source in the same way through the same place at no
     further charge.  You need not require recipients to copy the
     Corresponding Source along with the object code.  If the place to
     copy the object code is a network server, the Corresponding Source
     may be on a different server (operated by you or a third party)
     that supports equivalent copying facilities, provided you maintain
     clear directions next to the object code saying where to find the
     Corresponding Source.  Regardless of what server hosts the
     Corresponding Source, you remain obligated to ensure that it is
     available for as long as needed to satisfy these requirements.
 
     e) Convey the object code using peer-to-peer transmission, provided
     you inform other peers where the object code and Corresponding
     Source of the work are being offered to the general public at no
     charge under subsection 6d.
 
   A separable portion of the object code, whose source code is excluded
 from the Corresponding Source as a System Library, need not be
 included in conveying the object code work.
 
   A "User Product" is either (1) a "consumer product", which means any
 tangible personal property which is normally used for personal, family,
 or household purposes, or (2) anything designed or sold for incorporation
 into a dwelling.  In determining whether a product is a consumer product,
 doubtful cases shall be resolved in favor of coverage.  For a particular
 product received by a particular user, "normally used" refers to a
 typical or common use of that class of product, regardless of the status
 of the particular user or of the way in which the particular user
 actually uses, or expects or is expected to use, the product.  A product
 is a consumer product regardless of whether the product has substantial
 commercial, industrial or non-consumer uses, unless such uses represent
 the only significant mode of use of the product.
 
   "Installation Information" for a User Product means any methods,
 procedures, authorization keys, or other information required to install
 and execute modified versions of a covered work in that User Product from
 a modified version of its Corresponding Source.  The information must
 suffice to ensure that the continued functioning of the modified object
 code is in no case prevented or interfered with solely because
 modification has been made.
 
   If you convey an object code work under this section in, or with, or
 specifically for use in, a User Product, and the conveying occurs as
 part of a transaction in which the right of possession and use of the
 User Product is transferred to the recipient in perpetuity or for a
 fixed term (regardless of how the transaction is characterized), the
 Corresponding Source conveyed under this section must be accompanied
 by the Installation Information.  But this requirement does not apply
 if neither you nor any third party retains the ability to install
 modified object code on the User Product (for example, the work has
 been installed in ROM).
 
   The requirement to provide Installation Information does not include a
 requirement to continue to provide support service, warranty, or updates
 for a work that has been modified or installed by the recipient, or for
 the User Product in which it has been modified or installed.  Access to a
 network may be denied when the modification itself materially and
 adversely affects the operation of the network or violates the rules and
 protocols for communication across the network.
 
   Corresponding Source conveyed, and Installation Information provided,
 in accord with this section must be in a format that is publicly
 documented (and with an implementation available to the public in
 source code form), and must require no special password or key for
 unpacking, reading or copying.
 
   7. Additional Terms.
 
   "Additional permissions" are terms that supplement the terms of this
 License by making exceptions from one or more of its conditions.
 Additional permissions that are applicable to the entire Program shall
 be treated as though they were included in this License, to the extent
 that they are valid under applicable law.  If additional permissions
 apply only to part of the Program, that part may be used separately
 under those permissions, but the entire Program remains governed by
 this License without regard to the additional permissions.
 
   When you convey a copy of a covered work, you may at your option
 remove any additional permissions from that copy, or from any part of
 it.  (Additional permissions may be written to require their own
 removal in certain cases when you modify the work.)  You may place
 additional permissions on material, added by you to a covered work,
 for which you have or can give appropriate copyright permission.
 
   Notwithstanding any other provision of this License, for material you
 add to a covered work, you may (if authorized by the copyright holders of
 that material) supplement the terms of this License with terms:
 
     a) Disclaiming warranty or limiting liability differently from the
     terms of sections 15 and 16 of this License; or
 
     b) Requiring preservation of specified reasonable legal notices or
     author attributions in that material or in the Appropriate Legal
     Notices displayed by works containing it; or
 
     c) Prohibiting misrepresentation of the origin of that material, or
     requiring that modified versions of such material be marked in
     reasonable ways as different from the original version; or
 
     d) Limiting the use for publicity purposes of names of licensors or
     authors of the material; or
 
     e) Declining to grant rights under trademark law for use of some
     trade names, trademarks, or service marks; or
 
     f) Requiring indemnification of licensors and authors of that
     material by anyone who conveys the material (or modified versions of
     it) with contractual assumptions of liability to the recipient, for
     any liability that these contractual assumptions directly impose on
     those licensors and authors.
 
   All other non-permissive additional terms are considered "further
 restrictions" within the meaning of section 10.  If the Program as you
 received it, or any part of it, contains a notice stating that it is
 governed by this License along with a term that is a further
 restriction, you may remove that term.  If a license document contains
 a further restriction but permits relicensing or conveying under this
 License, you may add to a covered work material governed by the terms
 of that license document, provided that the further restriction does
 not survive such relicensing or conveying.
 
   If you add terms to a covered work in accord with this section, you
 must place, in the relevant source files, a statement of the
 additional terms that apply to those files, or a notice indicating
 where to find the applicable terms.
 
   Additional terms, permissive or non-permissive, may be stated in the
 form of a separately written license, or stated as exceptions;
 the above requirements apply either way.
 
   8. Termination.
 
   You may not propagate or modify a covered work except as expressly
 provided under this License.  Any attempt otherwise to propagate or
 modify it is void, and will automatically terminate your rights under
 this License (including any patent licenses granted under the third
 paragraph of section 11).
 
   However, if you cease all violation of this License, then your
 license from a particular copyright holder is reinstated (a)
 provisionally, unless and until the copyright holder explicitly and
 finally terminates your license, and (b) permanently, if the copyright
 holder fails to notify you of the violation by some reasonable means
 prior to 60 days after the cessation.
 
   Moreover, your license from a particular copyright holder is
 reinstated permanently if the copyright holder notifies you of the
 violation by some reasonable means, this is the first time you have
 received notice of violation of this License (for any work) from that
 copyright holder, and you cure the violation prior to 30 days after
 your receipt of the notice.
 
   Termination of your rights under this section does not terminate the
 licenses of parties who have received copies or rights from you under
 this License.  If your rights have been terminated and not permanently
 reinstated, you do not qualify to receive new licenses for the same
 material under section 10.
 
   9. Acceptance Not Required for Having Copies.
 
   You are not required to accept this License in order to receive or
 run a copy of the Program.  Ancillary propagation of a covered work
 occurring solely as a consequence of using peer-to-peer transmission
 to receive a copy likewise does not require acceptance.  However,
 nothing other than this License grants you permission to propagate or
 modify any covered work.  These actions infringe copyright if you do
 not accept this License.  Therefore, by modifying or propagating a
 covered work, you indicate your acceptance of this License to do so.
 
   10. Automatic Licensing of Downstream Recipients.
 
   Each time you convey a covered work, the recipient automatically
 receives a license from the original licensors, to run, modify and
 propagate that work, subject to this License.  You are not responsible
 for enforcing compliance by third parties with this License.
 
   An "entity transaction" is a transaction transferring control of an
 organization, or substantially all assets of one, or subdividing an
 organization, or merging organizations.  If propagation of a covered
 work results from an entity transaction, each party to that
 transaction who receives a copy of the work also receives whatever
 licenses to the work the party's predecessor in interest had or could
 give under the previous paragraph, plus a right to possession of the
 Corresponding Source of the work from the predecessor in interest, if
 the predecessor has it or can get it with reasonable efforts.
 
   You may not impose any further restrictions on the exercise of the
 rights granted or affirmed under this License.  For example, you may
 not impose a license fee, royalty, or other charge for exercise of
 rights granted under this License, and you may not initiate litigation
 (including a cross-claim or counterclaim in a lawsuit) alleging that
 any patent claim is infringed by making, using, selling, offering for
 sale, or importing the Program or any portion of it.
 
   11. Patents.
 
   A "contributor" is a copyright holder who authorizes use under this
 License of the Program or a work on which the Program is based.  The
 work thus licensed is called the contributor's "contributor version".
 
   A contributor's "essential patent claims" are all patent claims
 owned or controlled by the contributor, whether already acquired or
 hereafter acquired, that would be infringed by some manner, permitted
 by this License, of making, using, or selling its contributor version,
 but do not include claims that would be infringed only as a
 consequence of further modification of the contributor version.  For
 purposes of this definition, "control" includes the right to grant
 patent sublicenses in a manner consistent with the requirements of
 this License.
 
   Each contributor grants you a non-exclusive, worldwide, royalty-free
 patent license under the contributor's essential patent claims, to
 make, use, sell, offer for sale, import and otherwise run, modify and
 propagate the contents of its contributor version.
 
   In the following three paragraphs, a "patent license" is any express
 agreement or commitment, however denominated, not to enforce a patent
 (such as an express permission to practice a patent or covenant not to
 sue for patent infringement).  To "grant" such a patent license to a
 party means to make such an agreement or commitment not to enforce a
 patent against the party.
 
   If you convey a covered work, knowingly relying on a patent license,
 and the Corresponding Source of the work is not available for anyone
 to copy, free of charge and under the terms of this License, through a
 publicly available network server or other readily accessible means,
 then you must either (1) cause the Corresponding Source to be so
 available, or (2) arrange to deprive yourself of the benefit of the
 patent license for this particular work, or (3) arrange, in a manner
 consistent with the requirements of this License, to extend the patent
 license to downstream recipients.  "Knowingly relying" means you have
 actual knowledge that, but for the patent license, your conveying the
 covered work in a country, or your recipient's use of the covered work
 in a country, would infringe one or more identifiable patents in that
 country that you have reason to believe are valid.
 
   If, pursuant to or in connection with a single transaction or
 arrangement, you convey, or propagate by procuring conveyance of, a
 covered work, and grant a patent license to some of the parties
 receiving the covered work authorizing them to use, propagate, modify
 or convey a specific copy of the covered work, then the patent license
 you grant is automatically extended to all recipients of the covered
 work and works based on it.
 
   A patent license is "discriminatory" if it does not include within
 the scope of its coverage, prohibits the exercise of, or is
 conditioned on the non-exercise of one or more of the rights that are
 specifically granted under this License.  You may not convey a covered
 work if you are a party to an arrangement with a third party that is
 in the business of distributing software, under which you make payment
 to the third party based on the extent of your activity of conveying
 the work, and under which the third party grants, to any of the
 parties who would receive the covered work from you, a discriminatory
 patent license (a) in connection with copies of the covered work
 conveyed by you (or copies made from those copies), or (b) primarily
 for and in connection with specific products or compilations that
 contain the covered work, unless you entered into that arrangement,
 or that patent license was granted, prior to 28 March 2007.
 
   Nothing in this License shall be construed as excluding or limiting
 any implied license or other defenses to infringement that may
 otherwise be available to you under applicable patent law.
 
   12. No Surrender of Others' Freedom.
 
   If conditions are imposed on you (whether by court order, agreement or
 otherwise) that contradict the conditions of this License, they do not
 excuse you from the conditions of this License.  If you cannot convey a
 covered work so as to satisfy simultaneously your obligations under this
 License and any other pertinent obligations, then as a consequence you may
 not convey it at all.  For example, if you agree to terms that obligate you
 to collect a royalty for further conveying from those to whom you convey
 the Program, the only way you could satisfy both those terms and this
 License would be to refrain entirely from conveying the Program.
 
   13. Use with the GNU Affero General Public License.
 
   Notwithstanding any other provision of this License, you have
 permission to link or combine any covered work with a work licensed
 under version 3 of the GNU Affero General Public License into a single
 combined work, and to convey the resulting work.  The terms of this
 License will continue to apply to the part which is the covered work,
 but the special requirements of the GNU Affero General Public License,
 section 13, concerning interaction through a network will apply to the
 combination as such.
 
   14. Revised Versions of this License.
 
   The Free Software Foundation may publish revised and/or new versions of
 the GNU General Public License from time to time.  Such new versions will
 be similar in spirit to the present version, but may differ in detail to
 address new problems or concerns.
 
   Each version is given a distinguishing version number.  If the
 Program specifies that a certain numbered version of the GNU General
 Public License "or any later version" applies to it, you have the
 option of following the terms and conditions either of that numbered
 version or of any later version published by the Free Software
 Foundation.  If the Program does not specify a version number of the
 GNU General Public License, you may choose any version ever published
 by the Free Software Foundation.
 
   If the Program specifies that a proxy can decide which future
 versions of the GNU General Public License can be used, that proxy's
 public statement of acceptance of a version permanently authorizes you
 to choose that version for the Program.
 
   Later license versions may give you additional or different
 permissions.  However, no additional obligations are imposed on any
 author or copyright holder as a result of your choosing to follow a
 later version.
 
   15. Disclaimer of Warranty.
 
   THERE IS NO WARRANTY FOR THE PROGRAM, TO THE EXTENT PERMITTED BY
 APPLICABLE LAW.  EXCEPT WHEN OTHERWISE STATED IN WRITING THE COPYRIGHT
 HOLDERS AND/OR OTHER PARTIES PROVIDE THE PROGRAM "AS IS" WITHOUT WARRANTY
 OF ANY KIND, EITHER EXPRESSED OR IMPLIED, INCLUDING, BUT NOT LIMITED TO,
 THE IMPLIED WARRANTIES OF MERCHANTABILITY AND FITNESS FOR A PARTICULAR
 PURPOSE.  THE ENTIRE RISK AS TO THE QUALITY AND PERFORMANCE OF THE PROGRAM
 IS WITH YOU.  SHOULD THE PROGRAM PROVE DEFECTIVE, YOU ASSUME THE COST OF
 ALL NECESSARY SERVICING, REPAIR OR CORRECTION.
 
   16. Limitation of Liability.
 
   IN NO EVENT UNLESS REQUIRED BY APPLICABLE LAW OR AGREED TO IN WRITING
 WILL ANY COPYRIGHT HOLDER, OR ANY OTHER PARTY WHO MODIFIES AND/OR CONVEYS
 THE PROGRAM AS PERMITTED ABOVE, BE LIABLE TO YOU FOR DAMAGES, INCLUDING ANY
 GENERAL, SPECIAL, INCIDENTAL OR CONSEQUENTIAL DAMAGES ARISING OUT OF THE
 USE OR INABILITY TO USE THE PROGRAM (INCLUDING BUT NOT LIMITED TO LOSS OF
 DATA OR DATA BEING RENDERED INACCURATE OR LOSSES SUSTAINED BY YOU OR THIRD
 PARTIES OR A FAILURE OF THE PROGRAM TO OPERATE WITH ANY OTHER PROGRAMS),
 EVEN IF SUCH HOLDER OR OTHER PARTY HAS BEEN ADVISED OF THE POSSIBILITY OF
 SUCH DAMAGES.
 
   17. Interpretation of Sections 15 and 16.
 
   If the disclaimer of warranty and limitation of liability provided
 above cannot be given local legal effect according to their terms,
 reviewing courts shall apply local law that most closely approximates
 an absolute waiver of all civil liability in connection with the
 Program, unless a warranty or assumption of liability accompanies a
 copy of the Program in return for a fee.
 
                      END OF TERMS AND CONDITIONS
 
             How to Apply These Terms to Your New Programs
 
   If you develop a new program, and you want it to be of the greatest
 possible use to the public, the best way to achieve this is to make it
 free software which everyone can redistribute and change under these terms.
 
   To do so, attach the following notices to the program.  It is safest
 to attach them to the start of each source file to most effectively
 state the exclusion of warranty; and each file should have at least
 the "copyright" line and a pointer to where the full notice is found.
 
     <one line to give the program's name and a brief idea of what it does.>
     Copyright (C) <year>  <name of author>
 
     This program is free software: you can redistribute it and/or modify
     it under the terms of the GNU General Public License as published by
     the Free Software Foundation, either version 3 of the License, or
     (at your option) any later version.
 
     This program is distributed in the hope that it will be useful,
     but WITHOUT ANY WARRANTY; without even the implied warranty of
     MERCHANTABILITY or FITNESS FOR A PARTICULAR PURPOSE.  See the
     GNU General Public License for more details.
 
     You should have received a copy of the GNU General Public License
     along with this program.  If not, see <http://www.gnu.org/licenses/>.
 
 Also add information on how to contact you by electronic and paper mail.
 
   If the program does terminal interaction, make it output a short
 notice like this when it starts in an interactive mode:
 
     <program>  Copyright (C) <year>  <name of author>
     This program comes with ABSOLUTELY NO WARRANTY; for details type `show w'.
     This is free software, and you are welcome to redistribute it
     under certain conditions; type `show c' for details.
 
 The hypothetical commands `show w' and `show c' should show the appropriate
 parts of the General Public License.  Of course, your program's commands
 might be different; for a GUI interface, you would use an "about box".
 
   You should also get your employer (if you work as a programmer) or school,
 if any, to sign a "copyright disclaimer" for the program, if necessary.
 For more information on this, and how to apply and follow the GNU GPL, see
 <http://www.gnu.org/licenses/>.
 
   The GNU General Public License does not permit incorporating your program
 into proprietary programs.  If your program is a subroutine library, you
 may consider it more useful to permit linking proprietary applications with
 the library.  If this is what you want to do, use the GNU Lesser General
 Public License instead of this License.  But first, please read
 <http://www.gnu.org/philosophy/why-not-lgpl.html>.
 

Table of Contents
```
